# Metformin escape in prostate cancer by activating the PTGR1 transcriptional program through a novel super-enhancer

**DOI:** 10.1038/s41392-023-01516-2

**Published:** 2023-08-16

**Authors:** Jianheng Ye, Shanghua Cai, Yuanfa Feng, Jinchuang Li, Zhiduan Cai, Yulin Deng, Ren Liu, Xuejin Zhu, Jianming Lu, Yangjia Zhuo, Yingke Liang, Jianjiang Xie, Yanqiong Zhang, Huichan He, Zhaodong Han, Zhenyu Jia, Weide Zhong

**Affiliations:** 1 Department of Urology, Guangzhou First People’s Hospital, South China University of Technology, 510180 Guangzhou, Guangdong China; 2 Urology Key Laboratory of Guangdong Province, The First Affiliated Hospital of Guangzhou Medical University, Guangzhou Medical University, 510230 Guangzhou, Guangdong China; 3 Guangzhou National Laboratory, No. 9 XingDaoHuanBei Road, Guangzhou International Bio Island, 510005 Guangzhou, Guangdong China; 4 Institute of Chinese Materia Medica, China Academy of Chinese Medical Sciences, 100700 Beijing, China; 5 Department of Botany and Plant Sciences, University of California, Riverside, CA 92507 USA; 6 Graduate Program in Genetics, Genomics & Bioinformatics, University of California, Riverside, CA 92507 USA; 7 State Key Laboratory of Quality Research in Chinese Medicine, Macau University of Science and Technology, Taipa, 999078 Macau, China

**Keywords:** Urology, Urological cancer

## Abstract

The therapeutic efficacy of metformin in prostate cancer (PCa) appears uncertain based on various clinical trials. Metformin treatment failure may be attributed to the high frequency of transcriptional dysregulation, which leads to drug resistance. However, the underlying mechanism is still unclear. In this study, we found evidences that metformin resistance in PCa cells may be linked to cell cycle reactivation. Super-enhancers (SEs), crucial regulatory elements, have been shown to be associated with drug resistance in various cancers. Our analysis of SEs in metformin-resistant (MetR) PCa cells revealed a correlation with Prostaglandin Reductase 1 (PTGR1) expression, which was identified as significantly increased in a cluster of cells with metformin resistance through single-cell transcriptome sequencing. Our functional experiments showed that PTGR1 overexpression accelerated cell cycle progression by promoting progression from the G0/G1 to the S and G2/M phases, resulting in reduced sensitivity to metformin. Additionally, we identified key transcription factors that significantly increase PTGR1 expression, such as SRF and RUNX3, providing potential new targets to address metformin resistance in PCa. In conclusion, our study sheds new light on the cellular mechanism underlying metformin resistance and the regulation of the SE-TFs-PTGR1 axis, offering potential avenues to enhance metformin’s therapeutic efficacy in PCa.

## Introduction

Prostate cancer (PCa) is the most common male malignant tumor and the second leading cause of cancer death in the United States.^1^ To date, PCa therapy still faces great challenges due to the heterogeneous nature of the disease. Although PCa patients receiving androgen deprivation therapy (ADT), surgery, radiation and chemotherapy tend to have a lower risk of recurrence and better survival outcomes,^2^ manifestations of metabolic syndrome, such as obesity, insulin resistance and impaired glucose tolerance, often occur following treatment which eventually causes drug resistance and distal metastasis.^3^ To maintain a sufficient energy supply for PCa cells, alterations in cellular metabolism continue to occur during the progression from prostate intraepithelial neoplasia to metastasis. For example, healthy prostate cells utilize citrate to synthesize prostatic fluid so that the tricarboxylic acid (TCA) cycle is largely inhibited.^4^ In contrast, the energy production in aggressive PCa cells is accomplished mainly through the TCA cycle and oxidative phosphorylation (OXPHOS).^4^ However, OXPHOS level has been reported to be decreased while glycolytic activity is compensatorily increased in metastatic PCa cells.^5^ Taken together, the previous studies indicated that PCa may rely on distinct metabolic pathways for energy production at various stages, providing potential targets for precision therapy.

Metformin is a first-line oral hypoglycemic drug derived from extracts of the herb *Galega officinalis*. Accumulating studies have indicated that metformin may be a potential candidate adjuvant therapeutic agent for PCa due to its multiple anticancer effects, satisfactory tolerance in humans, and low cost.^6–8^ Metformin not only exerts excellent glucose-lowering effects, but also suppresses cancer growth by inducing G0/G1 arrest and decreasing OXPHOS level to modulate tumor cell metabolism.^7–9^ Although accumulating clinical data have demonstrated the association between metformin treatment and favorable outcomes in PCa patients,^10–14^ it has been reported that certain patients fail to respond to metformin.^15–18^ This is consistent with a previous study that preliminary demonstrated resistance to metformin treatment in various cancers due to tumor heterogeneity.^19^ Therefore, we hypothesized that a subpopulation of PCa patients may develop metformin resistance after a period of treatment and aimed to find insights into the underlying mechanism.

Specific phenotypes of a certain cancer, including acquired drug resistance stemming from gradual adaptation to extracellular stimulation, may be associated with unique genomic characteristics. Other studies have suggested that epigenetic alterations may be the precipitating factor of metformin resistance in different cancers.^19^ In addition, such alterations mainly occur in a preexisting cluster of cancer cells called drug-tolerant persister (DTP) cells,^20^ some of which may undergo reversible cell cycle arrest to acquire drug resistance.^21^ The proliferation of DTP cells can be activated from dormancy after exposure to anticancer agents through transcriptional rewriting programs.^20,21^ Young et al. identified clusters of extensive genomic domains occupied by master transcription factors and the mediator complex in embryonic stem cells (ESCs), which they named “super-enhancers” (SEs). These domains are distinctive due to their extensive DNA regions and strong correlation with the key identity of ESCs.^22^ SEs are usually identified using ChIP-seq and are characterized by an extremely high degree of enrichment of transcriptional coactivators, including Mediator (Med1), and chromatin marks.^23^ Recent studies have shown that SEs play a crucial role in maintaining cancer cell identity and are essential for resistance to multiple cancer treatments.^24–27^ In PCa, a novel SE activated by androgen receptor was identified as a potential cause of resistance to antiandrogen therapy due to its ability to abnormally activate choline metabolism.^25^ Therefore, we aimed to explore the role of SEs and their targeted genes in the develop of metformin resistance in PCa by using ChIP-seq and other experiments.

In this study, we generated two in vitro metformin-resistant (MetR) cell models using PCa cell lines, i.e., DU145 and 22RV1. Interestingly, after 30 days of drug withdrawal, these MetR cells regained metformin sensitivity, suggesting that metformin resistance may be a temporary nonmutational cell phenotype. Through our experiments and RNA-seq analysis, we found that metformin resistance in prostate cancer cells may be linked to changes in the cell cycle and metabolic reprogramming. Furthermore, our single-cell transcriptome sequencing results revealed that the key gene Prostaglandin Reductase 1 (PTGR1) was upregulated in a cluster of DU145-MetR cells which is associated with metformin resistance. H3K27ac ChIP-Seq data and other experiments indicated that a novel SE located upstream of PTGR1 and bound by the key transcription factors SRF and RUNX3 may be associated with the upregulation of PTGR1. We demonstrated that PTGR1 exerts an antagonizing effect on metformin treatment by interfering with cell cycle arrest which may be related to the role of E2F3. Our experiments for the first time showed that: (1) this SE increased PTGR1 expression in DU145-MetR cells when bound by SRF and RUNX3, and (2) elevated expression of PTGR1 accelerated cell cycle progression by promoting the progression from the G0/G1 to the S and G2/M phases, reducing the inhibitory effect of metformin on PCa.

## Results

### Development of metformin resistance in PCa cells after long-term treatment

**Fig. 1:**
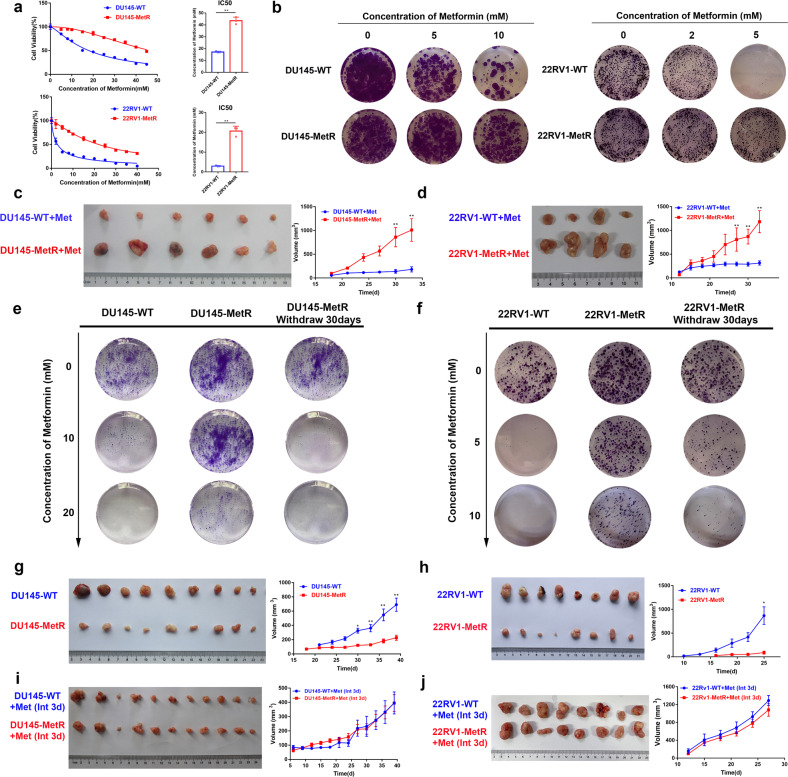
Prostate cancer cells acquire resistance to metformin after long-term treatment. **a** Representative metformin half-maximal inhibitory concentration (IC_50_) in two wild-type (WT) and metformin-resistant (MetR) prostate cancer cell lines (DU145, 22RV1, *n* = 3) determined by the CCK-8 assay and calculated by fitting a nonlinear regression curve. **b** Colony formation assay of DU145-WT, DU145-MetR, 22RV1-WT and 22RV1-MetR cells treated with different concentrations of metformin. **c**, **d** Nude mice (DU145 *n* = 6, 22RV1 *n* = 4) received continuous metformin treatment. Tumor volume growth curves and representative images of DU145 (**c**) and 22RV1 (**d**) tumors are shown. Metformin was administered at a concentration of 250 mg/kg. **e**, **f** Colony formation assay comparing the proliferation of WT cells, MetR cells and MetR cells after 30 days of metformin withdrawal. The results of DU145 cells (**e**) and 22RV1 cells (**f**) are shown. **g**, **h** Nude mice (DU145 *n* = 9, 22RV1 *n* = 8) were fed a diet without metformin. Tumor volume growth curves and representative images of DU145 (**g**) and 22RV1 (**h**) tumors are shown. **i**, **j** Nude mice (DU145 *n* = 11, 22RV1 *n* = 8) received metformin every 3 days. Tumor volume growth curves and representative images of DU145 (**i**) and 22RV1 (**j**) tumors are shown. Metformin was administered at a concentration of 250 mg/kg. The tumor sizes were measured at 3-day intervals as soon as the tumors were palpable. *P < 0.05, **P < 0.01, the error bar indicates the standard deviation

Although metformin has been demonstrated to have a cancer-prevention effect, how PCa cells respond to long-term metformin exposure has rarely been explored. Here, we first tried to construct the metformin-resistant PCa cell model (MetR) by continuously treating DU145 and 22RV1 cells, which have distinct characteristics and genetic backgrounds, with the corresponding half-maximal inhibitory concentration (IC_50_) of the agent. CCK-8 assays, clone formation assays, and studies in subcutaneous xenograft tumor models showed that treated DU145 cells and 22RV1 cells were resistant to metformin after a long period of exposure (Fig. 1a–d). Metformin resistance was suggested to be associated with transcriptional programs that may induce reversible cell cycle arrest.^19,21,28^ Our clone formation assay showed that MetR cells restored metformin sensitivity after thirty days of drug withdrawal (Fig. 1e, f), for example, the proliferation rate of these MetR cells eventually became similar to that of wild-type (WT) cells. These results indicated that metformin resistance in PCa cells is likely to be a transient phenotype. This is in agreement with previous studies, which indicated that metformin resistance may be associated with reversible cell cycle arrest.^19,21,28^ Furthermore, MetR cells (DU145-MetR or 22RV1-MetR) and the corresponding control cells (DU145-WT or 22RV1-WT) were paired and subcutaneously injected into the flanks of male nude mice, which were treated with daily feeding of a diet without metformin or a diet intermittently containing metformin every three days. The results showed that the mice injected with the MetR cells had significantly smaller tumor sizes than those injected with the WT cells (Fig. 1g, h). Interestingly, we observed no significant difference in tumor size between the resistant cell injected groups and the control groups in the intermittent metformin feeding model (Fig. 1i, j). Taken together, our data indicated that the development of metformin resistance in PCa cells is due to continuous stimulation by the agent.

### Metformin resistance in PCa is acquired through cell cycle reactivation and metabolic reprogramming

**Fig. 2:**
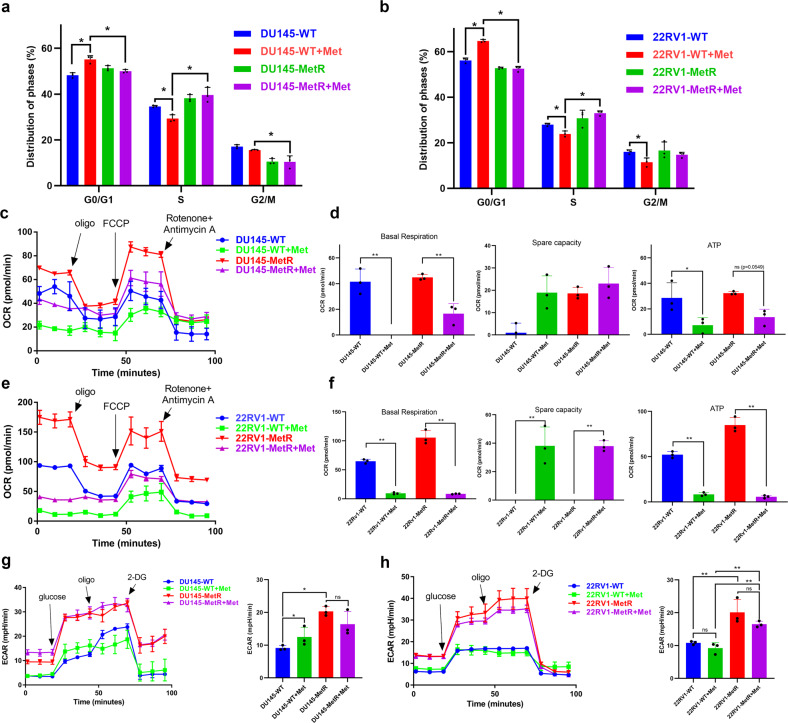
Reactivation of cell cycle progression and metabolic reprogramming contributes to metformin resistance in prostate cancer cells. **a** The percentages of G0/G1-, S-, and G2M-phase cells in each group of DU145 cells were determined by using flow cytometric analysis. Metformin (20 mM) was used in the treatment group. **b** The percentages of G0/G1-, S-, and G2M-phase cells in each group of 22RV1 cells were determined by using flow cytometric analysis. Metformin (10 mM) was used in the treatment group. **c**, **d** The oxygen consumption rate in DU145 cells treated with or without metformin (20 mM) was measured by Seahorse assay (**c**). Basal respiration, Spare respiratory capacity and ATP production were calculated (**d**). **e**, **f** The oxygen consumption rate in 22RV1 cells treated with or without metformin (10 mM) was measured by Seahorse assay (**e**). Basal respiration, Spare respiratory capacity and ATP production were calculated (**f**). **g** Extracellular acidification rate in each group of DU145 cells was measured by Seahorse assay. Metformin (20 mM) was used in the treatment group. **h** The extracellular acidification rate in each group of 22RV1 cells was measured by a Seahorse assay. Metformin (10 mM) was used in the treatment group. *n* = 3. Oligo oligomycin, FCCP carbonyl cyanide 4-trifluoromethoxy-phenylhydrazone, 2-DG 2-deoxy-d-glucose. *P < 0.05, **P < 0.01, the error bar indicates the standard deviation

As shown in Fig. 2a, b, metformin treatment induced cell cycle arrest in WT cells, as determined by the accumulation of G0/G1-phase cells, consistent with previous report.^29^ Furthermore, we found an increase in S-phase cells and a concomitant decrease in G0/G1-phase cells among the MetR cells, and such changes became more substantial after metformin treatment. However, there were no significant differences in cell invasion, migration and apoptosis between WT cells and MetR cells (Supplementary Fig. S1). On the other hand, we proved that metformin can effectively inhibit OXPHOS and the production of ATP in PCa in our recent study.^30^ Thus, we next sought to determine whether continued treatment with metformin would alter the primary metabolic pathways that maintain PCa cell growth. We quantified the level of OXPHOS and glycolytic activity by measuring the oxygen consumption rate (OCR) and extracellular acidification rate (ECAR) respectively using a Seahorse assay, showing that DU145-MetR cells exhibited a higher OCR than DU145-WT cells when treated with metformin (Fig. 2c). The inhibition of basal respiration and ATP production by metformin was much weaker in DU145-MetR cells than those in DU145-WT cells (Fig. 2d). However, basal respiration and ATP production were significantly suppressed in both 22RV1-MetR and 22RV1-WT cells when metformin was applied (Fig. 2e, f). Interestingly, the ECAR in both MetR cell lines were significantly increased regardless of metformin treatment, suggesting that PCa cells have the ability to activate glycolysis to compensate for their energy supply needs (Fig. 2g, h). Taken together, these results indicated that PCa cells may acquire metformin resistance through cell cycle reactivation and metabolic reprogramming.

### Relevance of aberrant activation of SEs and their target genes in PCa cells with metformin resistance

**Fig. 3:**
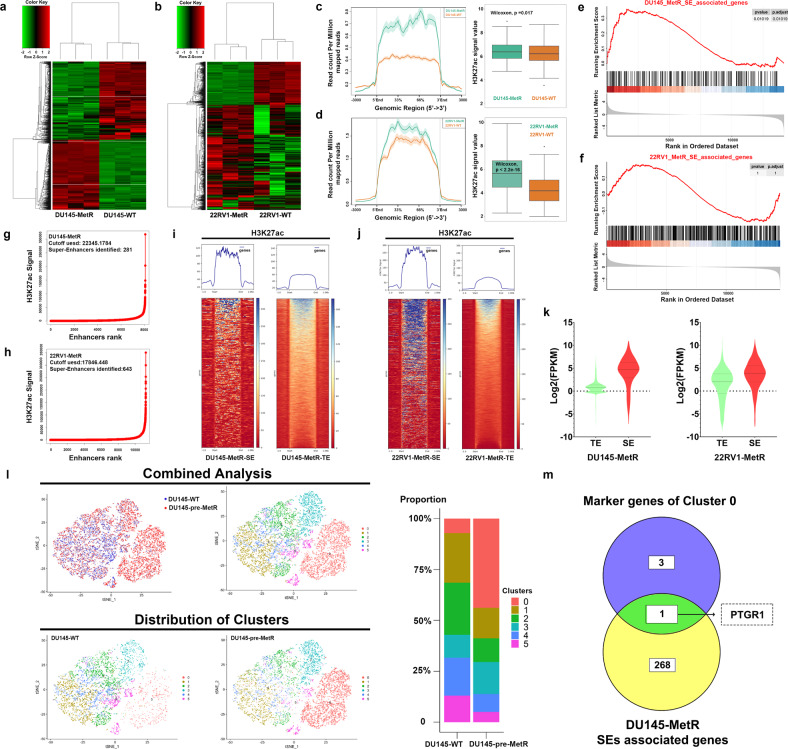
Aberrant activation of super-enhancer and its associated gene PTGR1 in a preexist cluster of prostate cancer cells may be associated with metformin resistance. **a** Heatmap of the RNA-seq results in DU145-WT and DU145-MetR cells (*n* = 3). Each row represents the transformed FPKM Z-score of an individual gene. **b** Heatmap of the RNA-seq results in 22RV1-WT and 22RV1-MetR cells (*n* = 3). Each row represents the transformed FPKM Z-score of an individual gene. **c** Average intensity curves of the H3K27ac ChIP-seq signal at the super-enhancer regions and the ±3 kb flanking regions in DU145-WT and DU145-MetR cells. Boxplot of super-enhancer peak density between DU145-MetR and DU145-WT cells. **d** Average intensity curves of H3K27ac ChIP-seq signal at the super-enhancer regions and the ±3 kb flanking regions in 22RV1-WT and 22RV1-MetR cells. Boxplot of super-enhancer peak density between 22RV1-MetR and 22RV1-WT cells. **e** GSEA analysis based on the pre-ranking genes that ordered by the fold change (FC) from differentially expressed analysis in DU145-MetR cells versus. DU145-WT cells with the input annotation generated from DU145-MetR SE-associated genes. **f** GSEA analysis based on the pre-ranking genes that ordered by the fold change (FC) from differentially expressed analysis in 22RV1-MetR cells versus. 22RV1-WT cells with the input annotation generated from 22RV1-MetR SE-associated genes. **g** DU145-MetR enhancer ranking plot based on H3K27ac ChIP-Seq signals using the ROSE algorithm. **h** 22RV1-MetR enhancer ranking plot based on H3K27ac ChIP-Seq signals using the ROSE algorithm. **i** Heatmaps of H3K27ac ChIP-seq signals at super-enhancer (left, SE) or typical enhancer (right, TE) regions in DU145-MetR cells. **j** Heatmaps of H3K27ac ChIP-seq signals at super-enhancer (left, SE) or typical enhancer (right, TE) regions in 22RV1-MetR cells. **k** Log2(FPKM) of typical enhancer-associated genes and super-enhancers-associated genes in DU145-MetR cells (left) and 22RV1-MetR cells (right). **l** T-distributed stochastic neighbor embedding (t-SNE) plot for the sub-clusters identified by single-cell RNA-sequencing analysis in DU145 pre-MetR and DU145-WT, and stacked bar chart for the distribution of each subcluster in DU145 pre-MetR and DU145-WT. **m** The gene PTGR1 was identified by intersection analysis of H3K27ac ChIP-Seq results and marker genes of Cluster 0

To understand the underlying mechanisms, we first conducted RNA-seq on metformin-resistant cell lines (Fig. 3a, b). Differential expression analysis revealed that 602 genes were significantly upregulated and 687 genes were downregulated in DU145-MetR cells compared to DU145-WT cells (Supplementary Fig. S2a) and that 996 genes were significantly upregulated and 406 genes were downregulated in 22RV1-MetR cells compared to 22RV1-WT cells (Supplementary Fig. S2b). The differentially expressed genes (DEGs) between DU145-MetR and DU145-WT cells were enriched in cell growth and metabolic pathways (sterol metabolic process, fatty acid metabolic process, and prostanoid metabolic process) (Supplementary Fig. S2c), while the DEGs between 22RV1-MetR and 22RV1-WT cells were enriched in the cell cycle and DNA replication pathways (Supplementary Fig. S2d). The fold changes in the expression levels of metabolic DEGs and cell cycle-related DEGs shown in detail in Supplementary Fig. S2e,f, indicating that cell cycle-related genes and metabolic genes were differentially expressed in metformin-resistant cells. Studies have demonstrated that SEs and their associated networks of transcription factors play an important role in regulating drug resistance and progression in prostate cancer.^25^ SEs are characterized by higher levels of binding of chromatin factors associated with enhancer activity, such as cohesin, and histone modifications, including H3K27ac, dimethylation of histone H3 at lysine 4 (H3K4me2), and H3K4me1.^22^

In our study, to test the hypothesis that the genes contributing to metformin resistance are upregulated by transcriptional programs such as histone modification and cis-regulatory elements, we performed H3K27ac ChIP-Seq in both WT and MetR cells. ChIP-Seq data were processed through ROSE (Rank Ordering of super-enhancers) to identify super-enhancers and typical enhancers (TEs).^31^ In order to access the statistical difference of H3K27ac signal in DU145-MetR SE region between DU145-MetR and DU145-WT cells, we quantified and normalized the average read coverage as the peak density of each DU145-MetR SE by using Homer annotatePeaks.pl function^32^ and presented the difference by boxplot in Fig. 3c, d. The boxplot indicated significant difference between DU145-MetR and DU145-WT cells (Wilcoxon, *P* value = 0.017) and a significant difference between 22RV1-MetR and 22RV1-WT cells (Wilcoxon, *P* value < 2.2e-16). The results showed that the H3K27ac signal in the super-enhancer regions of MetR cells was significantly stronger than that in WT cells (Fig. 3c, d). We next conducted gene set enrichment analysis (GSEA) based on the fold changes in the expression of preranked genes differentially expressed between DU145-MetR and DU145-WT cells. The input annotations were generated from the SE-associated genes in DU145-MetR cells. Our results showed that SEs associated genes signature enriched in DU145-MetR cells versus DU145-WT cells (Fig. 3e, NES = 1.43, adjusted *P* = 0.01). However, the SEs associated genes signature between 22RV1-MetR cells and 22RV1-WT cells showed no significant difference (Fig. 3f, NES = 0.73, adjusted *P* = 1).

With the ROSE algorithm, 281 SEs and 7788 TEs were called in DU145-MetR cells, while 643 SEs and 10,610 TEs were called in 22RV1-MetR cells (Fig. 3g, h). Moreover, 269 and 603 genes were annotated as SE-associated genes in DU145-MetR cells and 22RV1-MetR cells, respectively. The composite heatmap showed that, in both MetR cell lines, the increases in H3K27ac in SEs were significantly greater than that in TEs (Fig. 3i, j). Although the number of SEs was much less than that of TEs in MetR cells, the expression levels of SE-associated genes were significantly higher than those of genes associated with TEs (Fig. 3k). Taken together, our data indicated that aberrant activation of SEs and their target genes may account for metformin resistance in PCa cells.

### Upregulation of PTGR1 in PCa cells indicates metformin resistance

**Fig. 4:**
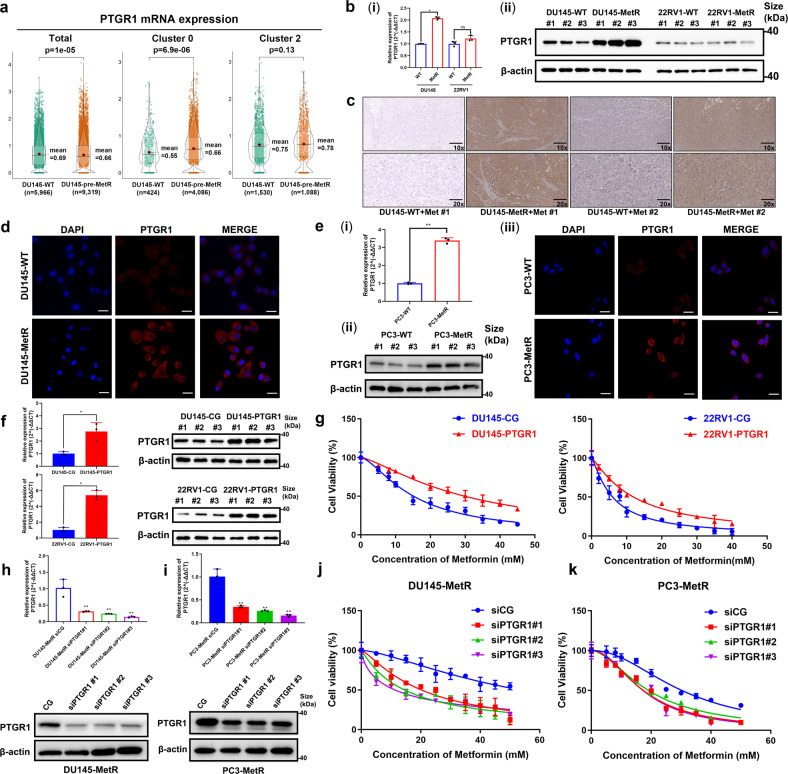
Increased expression of the super-enhancer-associated gene PTGR1 is associated with acquired metformin resistance. **a** Relative mRNA levels of PTGR1 in the general population (left), Cluster 0 (resistant) and Cluster 2 (sensitive) of DU145 pre-MetR cells from single-cell RNA sequencing (DU145 pre-MetR, cells undergoing metformin resistance). **b** mRNA (i) and protein (ii) levels of PTGR1 in DU145 and 22RV1 cells with or without metformin resistance were measured by qRT‒PCR and western blot analysis (*n* = 3). **c** Representative immunohistochemistry (IHC) images showing the PTGR1 expression pattern in the subcutaneous xenograft tissue from mice in the DU145-WT+Met and DU145-MetR+Met groups. Scale bar = 50 μm. **d** Immunofluorescence (IF) analysis of PTGR1 expression in DU145-WT and DU145-MetR cells. Representative images are shown. Scale bar = 20 μm. **e** mRNA (i) and protein (ii) levels of PTGR1 in PC3 cells with or without metformin resistance. (iii) IF analysis of PTGR1 expression in PC3-WT and PC3-MetR cells are shown. Scale bar = 20 μm. **f** qRT‒PCR and western blot analysis were utilized to validate the overexpression of PTGR1 in DU145 and 22RV1 cells transfected by lentiviral plasmids (*n* = 3). **g** The effect of PTGR1 overexpression on metformin treatment in DU145 (20 mM) and 22RV1 (10 mM) cells was analyzed by a CCK-8 assay (*n* = 3). **h**, **i** PTGR1 expression was decreased in DU145-MetR cells and PC3-MetR cells using siRNA, and the effect was validated by qRT‒PCR and western blot analysis. **j**, **k** The effect of decreased expression of PTGR1 on metformin treatment in DU145-MetR (20 mM) and PC3-MetR (20 mM) cells was analyzed by a CCK-8 assay (*n* = 3). *P < 0.05, **P < 0.01, the error bar indicates the standard deviation

The androgen receptor (AR) has been demonstrated to have an impact on the effectiveness of metformin treatment, and typically, drug resistance develops in only a subset of cancer cells, not all cells.^20,22^ To explore whether a subgroup of DU145 cells with AR-negative expression is predisposed to acquiring metformin resistance, we performed single-cell RNA-seq analysis on a DU145 cell model undergoing the acquisition of metformin resistance (DU145 pre-MetR) and identified 6 clusters (0 through 5) using unsupervised clustering (Fig. 3l). Our comparison with DU145-WT cells revealed that the number of cells in Cluster 0 was increased by more than 30%, while the numbers of cells in the other clusters were decreased (Table 1). We designated the cells in Cluster 0 as DTP-like cells, which could potentially develop metformin resistance. Four marker genes were identified within Cluster 0. By intersecting these data with the H3K27ac ChIP-Seq data, we found a common gene, Prostaglandin Reductase 1 (PTGR1) between the marker genes of Cluster 0 and the SE-associated genes in DU145-MetR cells (Fig. 3m), in addition, mRNA level of PTGR1 was increased in Cluster 0 (Fig. 4a). Notably, among the four marker genes (PTGR1, DDIT4, CEBPD, and EEF1A1) associated with the metformin-resistant subcluster (Cluster 0), only PTGR1 was significantly positively correlated with the cell cycle in the TCGA-PRAD dataset (Supplement Fig. S2g).

**Table 1:** Cell numbers of each cluster in WT group and pre-MetR group of DU145 cells

Cluster	WT	pre-MetR	Trend (%)
0	424	4086	36.74
1	1453	1392	−9.42
2	1530	1088	−13.97
3	670	1466	4.50
4	1113	818	−9.88
5	776	469	−7.97
Total	5966	9319	

*WT wild-type* cells, *pre-MetR* cells undergoing metformin resistance

Trend (%): Cluster (pre-MetR)/Total (pre-MetR)-Cluster (WT)/total (WT) × 100%

Next, by assessing its expression at both the mRNA and protein levels, we confirmed that PTGR1 was significantly upregulated in DU145-MetR cells compared with DU145-WT cells (Fig. 4b–d). However, we did not observe any difference in PTGR1 expression between 22RV1-WT cells and 22RV1-MetR cells (Fig. 4b), consistent with the assumption that the metformin efficacy is somehow compromised or confounded in AR-positive PCa cells. To gain further insights, we expanded our analysis by constructing another metformin-resistant cell model with PC3 cells (PC3-MetR), another AR-negative PCa cell line. The results of qRT‒PCR, western blot analysis, and immunofluorescence analysis showed that PTGR1 was overexpressed in PC3-MetR cells compared to PC3-WT cells (Fig. 4e). To assess the presumed relationship between PTGR1 expression and metformin efficacy, we applied lentiviral transduction to establish two PCa cell lines, i.e., DU145-PTGR1 and 22RV1-PTGR1, that can stably express PTGR1 (Fig. 4f). The high level of PTGR1 expression was found to significantly attenuate metformin efficacy in both DU145 and 22RV1 cells (Fig. 4g). Moreover, when we downregulated PTGR1 by transiently transfecting MetR cells with siRNA, the effect of metformin was enhanced as expected (Fig. 4h–k). In summary, our data and results implied that increased expression of PTGR1 in PCa indicates metformin resistance and that PTGR1 may serve as a biomarker for metformin treatment selection.

### Upregulation of PTGR1 promotes cell cycle progression and is related to poor survival in PCa patients

**Fig. 5:**
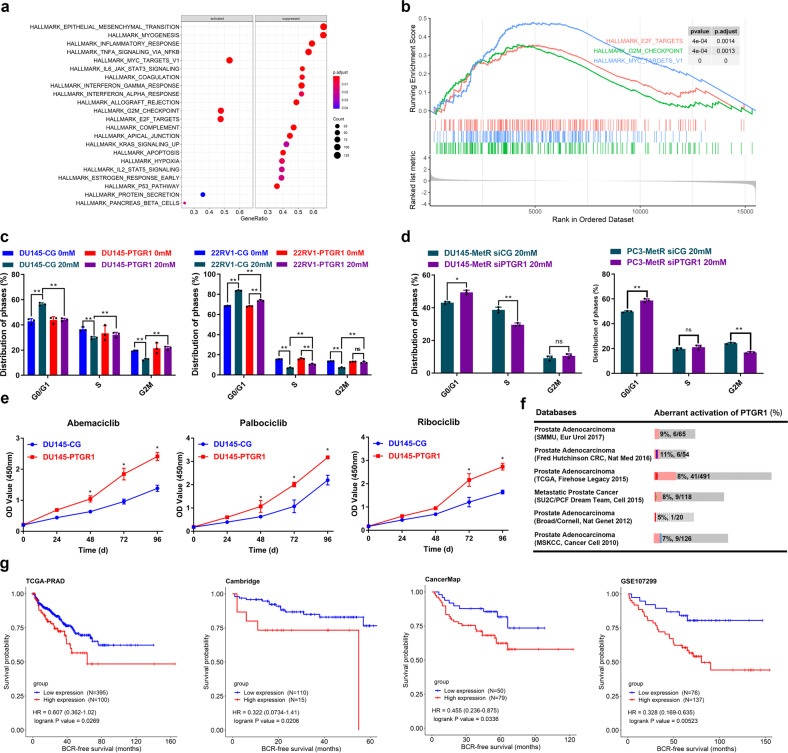
Upregulation of PTGR1 promoted cell cycle progression and was associated with poor survival in prostate cancer patients. **a** Bubble plot shows the biological pathways activated/suppressed in the high PTGR1 expression group. **b** Gene set enrichment analysis (GSEA) plot of cell cycle-related pathways enriched in the high PTGR1 expression group. **c** The effect of PTGR1 overexpression on the cell cycle with or without metformin treatment (20 mM) was analyzed by flow cytometry analysis. The percentages of G0/G1-, S-, and G2M-phase cells were compared among the groups. (*n* = 3) **d** The effect of decreased expression of PTGR1 on the cell cycle with metformin treatment (20 mM) was analyzed by flow cytometry analysis. The percentages of G0/G1-, S-, and G2M-phase cells were compared among the groups. (*n* = 3) **e** The effect of PTGR1 overexpression on CDK4/6 inhibitors, including abemaciclib, palbociclib and ribociclib, was analyzed by a CCK-8 assay. The concentration of each CDK4/6 inhibitor was 10 nM. Optical density values were determined by CCK-8 assay and measured at 450 nm (*n* = 3). **f** The proportion of PCa patients with aberrant activation of PTGR1 was analyzed in public databases by cBioPortal (http://www.cbioportal.org/). **g** Biochemical recurrence (BCR)-free survival of patients with high PTGR1 expression was compared with that of patients with low PTGR1 expression in the TCGA and GEO databases. *P < 0.05, **P < 0.01, the error bar indicates the standard deviation

To investigate how PTGR1 antagonizes metformin treatment, GSEA was employed to delineate the potential biological pathways involving PTGR1 in PCa. The results showed that cell cycle-related pathways, i.e., the MYC, G2M checkpoint, and E2F target pathways, were enriched in the PTGR1-upregulated group, indicating the relatedness between the antagonizing effect of PTGR1 on metformin treatment and the activation of cell cycle pathways (Fig. 5a, b). We then utilized flow cytometric analysis to demonstrate that upregulated PTGR1 can effectively abrogate metformin-induced G0/G1 arrest and promote S and G2/M-phase entry in cancer cells (Fig. 5c). In contrast, downregulated PTGR1 restored metformin sensitivity in MetR cells by blocking cells in G0/G1 phases and decreasing the population of cells in S phase or G2/M-phase (Fig. 5d). To investigate the impact of PTGR1 on the cell cycle, we performed western blot analysis. Our results showed that the expression of E2F Transcription Factor 3 (E2F3) was upregulated in DU145-MetR cells and in DU145 cells that stably overexpressed PTGR1. Furthermore, when PTGR1 expression was suppressed through siRNA transfection in DU145 cells, the expression of E2F3 was downregulated (Supplementary Fig. S3a). It is known that upregulation of E2F3 plays a crucial role in promoting the S-G2 transcriptional program.^33^ Therefore, we treated DU145-PTGR1 cells with CDK4/6 inhibitors, including Ribociclib, Palbociclib, and Abemaciclib. These inhibitors have been shown to induce G1 arrest by targeting the Retinoblastoma/E2F repressive complex.^34^ Our results showed that the group with high expression of PTGR1 exhibited a higher growth rate than the control group (Fig. 5e). These data and results suggested that PTGR1 likely exerts an antagonizing effect on metformin treatment by interfering with cell cycle arrest, which may be related to the role of E2F3.

In the literature, high expression of PTGR1 has been reported to be associated with poor prognosis in several types of cancers.^35^ To assess the clinical significance of PTGR1 in PCa, we surveyed its expression in PCa patients across public databases. For each PCa cohort in Fig. 5f, aberrant activation of PTGR1 was frequently found in a subset of patients who may show no response to metformin treatment. Further exploration of The Cancer Genome Atlas (TCGA) and Gene Expression Omnibus (GEO) databases indicated that PCa patients with high PTGR1 expression had shorter biochemical recurrence (BCR)-free survival times than those with low PTGR1 expression (Fig. 5g).

### PTGR1 expression is upregulated by super-enhancer bound by the master transcription factors SRF and RUNX3

**Fig. 6:**
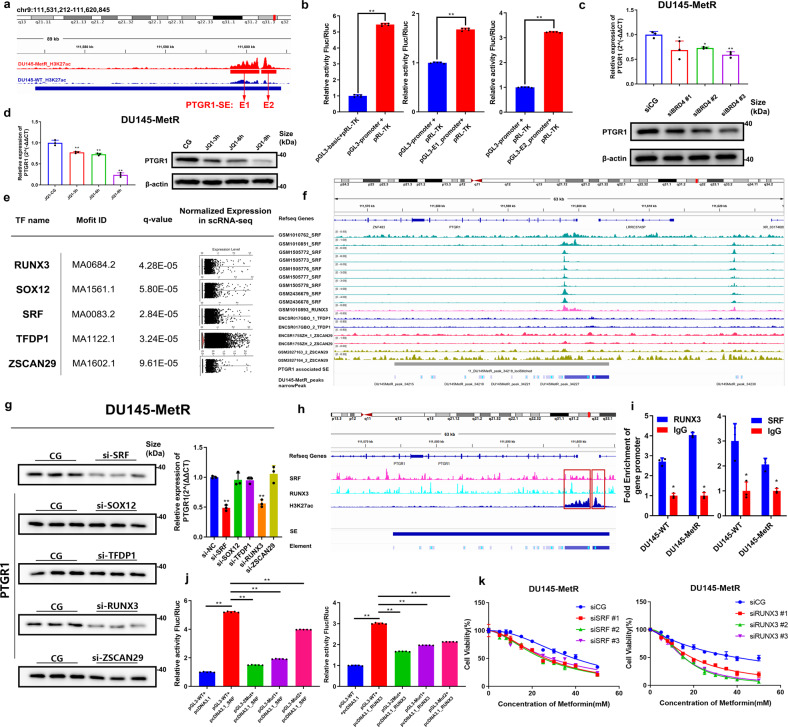
PTGR1 is upregulated by an upstream super-enhancer bound by the master transcription factors SRF and RUNX3. **a** Genome browser view of normalized H3K27ac ChIP-seq signals at the PTGR1 locus in DU145-WT and DU145-MetR cells. Two tracks were the average of two biological replicates. The super-enhancer (SE) region is marked by the blue line. The two constituent enhancers (Element 1, E1 and Element 2, E2) within the PTGR1-SE region are marked by the red lines. **b** Luciferase reporter assays were performed in 293T cells to validate the combination of SE and PTGR1. The Luciferase signal was normalized to the Renilla transfection control luciferase signal (*n* = 5). **c** qRT-PCR and western blot analysis were used to evaluate the expression level of PTGR1 in DU145-MetR cells with downregulation of BRD4 (*n* = 3). **d** qRT-PCR and western blot analysis were used to evaluate the expression level of PTGR1 in DU145-MetR cells treated with 1 μM JQ1 (*n* = 3). **e** Summary of TF motif occurrences within E1 and E2 elements. TFs expression in the metformin resistance cluster is shown in the dot plot for each motif. Statistically significant motif matches identified by FIMO were defined as *P* value (i.e., *q* value) <0.05. **f** Capture of the The Cistrome Data Browser showed the locations of the predicted TFs binding sites and the PTGR1-SE locus. **g** qRT-PCR and western blot analysis were performed to assess the mRNA and protein levels of PTGR1 after downregulating the predicted TFs (*n* = 3). The corresponding protein bands representing beta-actin are shown in Supplementary Fig. S5. **h** The binding sites indicated by the SRF ChIP-seq and RUNX3-ChIP-seq. **i** ChIP-qPCR analysis for enrichment of RUNX3 and SRF at the super-enhancer identified in Fig. 6a (*n* = 3). **j** Luciferase reporter assays were performed in 293T cells to validate the combination of SE with SRF and RUNX3, respectively. The Luciferase signal was normalized to the Renilla transfection control luciferase signal (*n* = 5). **k** The effect of decreased expression of SRF (left) or RUNX3 (right) on metformin treatment was analyzed by CCK-8 assay (*n* = 3). *P < 0.05, **P < 0.01, error bar indicates the standard deviation

To understand the relationship between PTGR1 expression and metformin resistance in DU145-MetR cells, we next investigated the underlying mechanism. super-enhancers (SEs) are critical regulatory elements and have been linked to the expression of genes associated with drug resistance.^24–26^ Our hypothesis was that the upregulation of PTGR1 in DU145-MetR cells might be associated with a specific SE. Thus, we conducted H3K27ac ChIP-Seq and applied the ROSE algorithm (Rank Ordering of super-enhancers), which is based on the active enhancer marker histone modification H3K27ac. Our analysis revealed a total of 281 SEs in DU145-MetR cells, one of which was located ~10 kb upstream of the transcription start site (TSS) of PTGR1. We selected the two constituent enhancers with the highest intensity signals and named them E1 and E2 (Fig. 6a). To examine the regulatory function of this SE on PTGR1 expression, we constructed two plasmids that contained minimal promoters of E1 and E2 to drive luciferase expression, and then transfected these plasmids into 293T cells. The results showed that the transcriptional activity was increased when either the E1 or E2 plasmid was successfully transfected and was significantly higher in cells with E2 transfection (Fig. 6b).

To further confirm that the SE specifically regulates PTGR1, we also evaluated the expression of six neighboring genes located within a 20-kb flanking distance from the PTGR1-SE site: DNAJC2, GNG10, ECPAS, LRRC37A5P, SHOC1, and ZNF48. However, our analysis showed that the expression of these neighboring genes did not show statistically significant differences between DU145-MetR cells and DU145-WT cells (Supplementary Fig. S4a). It has been recognized that BRD4 is enriched in SEs, which is required for SE-regulated transcriptional activity.^36^ Therefore, we applied BRD4 siRNAs as well as JQ1, a BRD4 inhibitor, to impair the regulatory function of SEs in DU145-MetR cells. As expected, the expression of PTGR1 was significantly decreased at both the mRNA and protein levels after BRD4 downregulation with siRNAs, and the experiment with JQ1 produced consistent results (Fig. 6c, d). However, the expression levels of these six neighboring genes did not exhibit significant changes (Supplementary Fig. S4b, c).

Next, we sought to identify the transcription factors involved in the regulation of PTGR1 by the SE. To this end, we conducted motif analysis using Homer and scanned the motifs from the JASPAR 2022 database using FIMO with default parameters.^37^ We then explored the expression of the predicted TFs, including SRF, TFDP1, SOX12, ZSCAN29, and RUNX3, in the metformin-resistant cell cluster using single-cell RNA-Seq (Fig. 6e). We also obtained ChIP-Seq data of genomic regions enriched with these transcription factors and mapped their binding sites in the E1/E2 element using FIMO software, resulting in the identification of four transcription factors, SRF, RUNX3, TFDP1, and ZSCAN29 (Fig. 6f). Furthermore, qRT-PCR and western blot analyses revealed that PTGR1 expression was significantly reduced when SRF and RUNX3 were downregulated (Fig. 6g and Supplementary Fig. S5). Moreover, ChIP-seq analysis results showed that SRF and RUNX3 were able to bind to the SE regions of PTGR1 in DU145-MetR cells (Fig. 6h). To validate the ChIP-seq results, ChIP-qPCR was performed to quantify the occupancy of RUNX3 and SRF, and their enrichment was confirmed at the SE regions of PTGR1 (Fig. 6i). We then applied the luciferase reporter assay to evaluate the functionality of SRF and RUNX3 in the regulation of PTGR1 SE, and the results demonstrated that upregulation of SRF or RUNX3 can significantly increase luciferase expression (Fig. 6j). Notably, DU145-MetR cells became sensitive to metformin when SRF or RUNX3 was downregulated (Fig. 6k). Furthermore, we suppressed the expression of RUNX3 and SRF in DU145-MetR cells using transient transfection of siRNAs. The results showed that the expression levels of those six neighboring genes remained unchanged (as indicated in Supplementary Fig. S4d). Considering these results collectively, we concluded that SE activates PTGR1 expression by interacting with the key transcription factors SRF and RUNX3.

## Discussion

In recent decades, metabolic modifications have been recognized as cancer hallmarks and potential therapeutic targets to overcome factors related to clinical treatment failure, such as drug resistance.^38,39^ Although many metabolic therapies have shown positive effects in basic studies,^4,5^ very few of them have been approved by rigorous validation to enter the clinical phase. Considering its satisfactory tolerance in humans, metformin has recently been repurposed as a new adjuvant therapy in PCa management.^6–8^ Metformin can directly inhibit complex I in the electron transport chain to induce the phosphorylation of Adenosine Monophosphate-Activated Protein Kinase (AMPK), which consequently inhibits the PI3K/AKT/mTOR pathway, gluconeogenesis, and OXPHOS.^6–8^ In addition, metformin was found to not only interfere with androgen signaling, but also reduce the side effects of ADT.^40^ However, the clinical effect of metformin on PCa therapy appears to be uncertain. Elgendy et al. demonstrated that metformin treatment combined with hypoglycemia can significantly inhibit tumor growth by modulating the PP2A/GSK3b/MCL-1 axis.^41^ Their findings revealed a mechanism through which cancer cells can escape the effects of metformin treatment. Furthermore, another study demonstrated that metformin resistance is widespread in various cancer cell lines, a characteristic that may be related to the genetic background of these cells.^19^ Similarly, we found that PCa cells acquired metformin resistance after long-term treatment. Based on our observation of metformin use in the clinic, we hypothesized that the acquisition of drug resistance is generally one of the main causes of the discrepant clinical results.

Studies have demonstrated that cancer cells undergo reversible cell cycle arrest to adapt to the treatment environment, leading to drug resistance.^20,21^ We observed that the metformin sensitivity of MetR cells was restored after thirty days of drug withdrawal, likely due to the effect of metformin on cell cycle arrest. Interestingly, we found a large proportion of MetR cells in S and G2/M phases, suggesting that metformin resistance in PCa cells may be attributed to cell cycle reactivation from arrest. The literature shows that acquired metformin resistance may be attributed to transcriptome reprogramming,^28^ and resistance-related epigenetic alterations usually require transcriptome reprogramming that affects the binding of transcription factors and histone modifications.^42,43^ Indeed, our data indicated that transcriptional regulation appeared to be more active in MetR cells than in WT cells. Regulatory element enhancers may play an important role in drug resistance acquisition, since we found that they were enriched and associated with histone modification patterns that govern the expression of cell-type-specific genes.^43,44^ Notably, a relatively large group of enhancers called super-enhancers (SEs) was defined in 2013, and cell malignancy is often associated with changes in the transcriptional programs driven by SEs.^22,45^ However, no studies have focused on the role of SEs in influencing the efficacy of PCa treatment, however, one study identified the aberrant activation of a group of SEs in a PCa cell model of enzalutamide resistance.^25^ Specifically, SEs can upregulate the expression of Choline Phosphotransferase 1 (CHPT1), which is independent of the androgen receptor (AR) pathway, and then activate choline metabolism to confer enzalutamide resistance on castration-resistant PCa cells. In our study, aberrant activation of a number of SEs was found in DU145-MetR and 22RV1-MetR cells. As a result, the SE-associated genes were expressed at much higher levels than the genes regulated by TEs, suggesting that resistance is caused mainly by SEs and their downstream transcriptional programs. In addition, we found different effects in DU145 cells than in 22RV1 cells, a discrepancy that may be attributed to AR regulation. Thus, we chose DU145 and PC3 cells, which are AR-negative, for subsequent investigation so that potential AR interference could be prevented.

It has been reported that a preexisting cluster of cancer cells, which are characterized as slow-cycling or dormant, has the ability to escape from the effects of antiproliferative agents.^20,46^ An unresolved question is whether acquired metformin resistance in PCa is also initiated in a unique cluster of cells. Although the mechanisms of metformin resistance in PCa have been investigated,^19,28^ these studies analyzed the genome changes or modifications in the total cancer cell population as a whole, neglecting differences between cells. In recent years, single-cell RNA-Seq analysis has been well adopted to explore the responses of cancer cells to drugs and the mechanism underlying drug resistance. For example, a single-cell assay was performed to track the evolution trajectory during chemotherapy in triple-negative breast cancer, and the study identified a number of chemoresistant genes that were being significantly upregulated after neoadjuvant chemotherapy.^47^ Notably, these identified transcriptional programs that conferred chemo-resistance were not intrinsic but were initiated in only a fraction of cells upon treatment. Likewise, Taavitsainen et al. identified a cluster of LNCap cells that is mainly responsible for the development of enzalutamide resistance. They confirmed that the marker genes in this cell cluster are partially regulated by chromatin structure and transcriptional reprogramming.^48^ Similarly, our single-cell RNA-Seq analysis identified a cluster of DU145-pre-MetR cells with a higher proliferation rate post-continuous treatment with metformin, while the cell numbers in the other clusters were substantially reduced. We then combined the results of H3K27ac ChIP-Seq and single-cell RNA-Seq to detect PTGR1, potentially upregulated by an upstream SE, in this resistant cluster. By the binding of master transcription factors (TFs), RNA Polymerase II, the Mediator complex and BRD4, SEs can enhance the expression levels of downstream genes. The binding sites of TFs depend on SEs, and they are changeable due to external stimulation, allowing the maintenance and growth of cancer cells.^36^ Finally, we screened two TFs involved in the regulation of PTGR1, i.e., SRF and RUNX3, whose roles in promoting cancer development have been well-established in recent decades.^49,50^

PTGR1 belongs to the medium-chain dehydrogenase/reductase superfamily and plays a vital role in regulating the arachidonic acid metabolism pathway.^35^ Accumulating evidence indicates that PTGR1 is overexpressed in many cancer types, including lung cancer, breast cancer, gastric cancer, pancreatic cancer and liver cancer, and is associated with poor prognosis.^35^ Our bioinformatics analysis revealed that PCa patients with high PTGR1 expression had shorter biochemical recurrence (BCR)-free survival times than those with low PTGR1 expression. Some cancer studies have preliminarily explored the role of PTGR1 in regulating the cell cycle and resisting oxidative stress.^51,52^ Decreased PTGR1 expression in DU145 and PC3 cells induced G0/G1-phases arrest, which was related to the increased protein levels of p21, Caspase 3, and cleaved PARP, as well as the decreased expression of CCND1.^52^ Interestingly, metformin has been well recognized as an antiproliferative agent that induces cell cycle arrest. Our data showed that the expression of PTGR1 was increased in metformin-resistant DU145 and PC3 cells, whereas it was decreased by metformin withdrawal in a time-dependent manner (Supplementary Fig. S6). Upregulation of PTGR1 in both DU145 and 22RV1 cells can significantly attenuate the efficacy of metformin treatment by promoting S- and G2/M-phase entry.

**Fig. 7:**
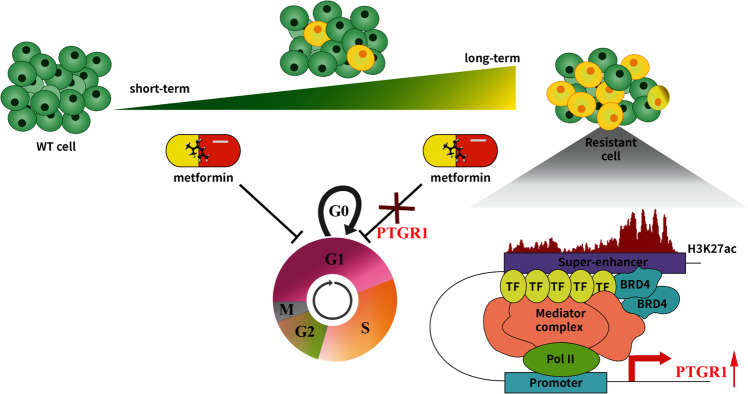
Schematic diagram showing that the SE-TFs-PTGR1 axis contributes to metformin resistance in PCa in a time-dependent manner

We present a novel finding that links the activation of the SE-TFs-PTGR1 axis with metformin resistance in PCa. Our data suggest that SE, through its interaction with the master transcription factors SRF and RUNX3, upregulates the expression of PTGR1 in the resistant cell cluster, thereby reducing the efficacy of metformin treatment by promoting cell cycle progression from the G0/G1 to the S and G2/M phases (Fig. 7). Our analysis of public datasets supports the clinical significance of our findings, as high expression of PTGR1 has been associated with a poor prognosis in PCa patients. These results highlight the importance of: (1) using metformin in an intermittent manner to prevent the development of resistance, and (2) exploring adjuvant therapies that target the SE/TFs/PTGR1 axis, potentially for use in combination with metformin. Further research is warranted to gain a deeper understanding of the metabolic changes underlying the development of metformin resistance in PCa, including the role of androgen receptor pathways.

## Materials and methods

### Construction of metformin-resistant prostate cancer cells

The prostate cancer cell lines DU145, PC3, and 22RV1 were used to construct metformin-resistant cell models. All human cancer cell lines were purchased from the American Type Culture Collection (ATCC) (Manassas, VA, USA) and cultured with their corresponding medium. We first calculated the half-maximal inhibitory concentration (IC_50_) of metformin in each cancer cell line at 72 h using the Cell Counting Kit-8 (CCK-8) assay. Then, 50,000 cancer cells were seeded in each well of six-well plates and cultured with the IC_50_ of metformin for 72 h. After that, ~50% of the cells in each well were transferred to culture dishes and cultured with half of the IC_50_ of metformin for one month. To construct the preresistant PCa cell model, 50,000 DU145 cells were cultured with the IC_50_ of metformin for 72 h and then cultured with half of the IC_50_ of metformin for 2 weeks. Finally, the resistance phenotype of the cell models was verified by a CCK-8 assay and in subcutaneous xenograft tumor models.

### Establishment of subcutaneous xenograft tumor model

Animal experiments were performed in compliance with the guidelines of the Animal Ethics Committee at South China University of Technology (Guangzhou, Guangdong, China). All BALB/c nude mice were divided into three groups: ① non-metformin feeding group; ② metformin feeding group (250 mg/kg metformin diluted in the drinking water); and ③ intermittent feeding group (metformin administration at intervals of 3 days). A total of 1 × 10^6^ cells from the control and MetR groups were injected into the left and right flank, respectively, of each BALB/c nude mouse. The tumor volume was calculated using the following formula: volume (mm^3^) = width^2^ (mm^2^) × length (mm)/2.

### Cell lines construction and transfection

DU145 and 22RV1 cells were infected with lentivirus containing the PTGR1 overexpression plasmid with a puro cassette and GFP tag. Stable cell lines were selected via growth in medium containing 3 g/mL puromycin 2 days after transfection. The targeting siRNA and negative control siRNA and the transfection reagent were obtained from GenePharma. The siRNA sequences are provided in the Supplementary. The transfection efficacy was tested by western blot analysis 72 h after transfection. Both constructed cell lines were confirmed by western blot analysis. The sequences are shown in the Supplementary File.

### Seahorse assay

A total of 8000 DU145 cells and 16000 22RV1 cells were seeded in each well of an XFe24 cell culture microplate and treated with medium with or without metformin for one day before the assay was performed. Mitochondrial function was determined by measuring the oxygen consumption rate using an XF Cell Mito Stress Test Kit (Agilent Technologies) according to the protocol of our previous study.^30^ Glycolytic activity was determined by measuring the extracellular acidification rate using an XF Glycolysis Stress Test Kit. The results were analyzed using Wave 2.6.0 software (Seahorse Bioscience). The Seahorse XF Cell Mito Stress Test Kit and XF Glycolysis Stress Test Kit were purchased from Agilent Technologies.

### ChIP-Seq and analysis

#### ChIP-Seq sample preparation & sequencing

H3K27ac, RUNX3, and SRF ChIP-Seq were performed using the EpiTM chromatin immunoprecipitation kit (Epibiotek, cat. no. R1802). First, a total of 2 × 10^6^ cells were collected and subjected to cross-linking with one percent formaldehyde for 10 min, and the reaction was then quenched with 0.125 M glycine for 5 min. To isolate nuclei, 1 mL lysis buffer was added, and the cell debris was collected by centrifugation at 2400 × *g* and 4 °C for 10 min. After that, nuclei were located in the supernatant and subjected to enzymatic shearing to generate chromatin fragments of an average length of between 200 and 500 bp by incubation at 37 °C for 10 min. The supernatant was collected by centrifugation at 18,000 × *g* and 4 °C for 10 min. The supernatant was mixed with the ChIP reaction mix (protein A/G magnetic beads, ChIP IP buffer, antibody, protease inhibitor cocktail) and incubated with rotation at 4 °C overnight. On the second day, after washing and removing the protein A/G magnetic beads from the mixture, the chromatin was eluted in reverse cross-linking buffer at 65 °C for 3 h. Next, the ChIP DNA was mixed with RNase A and protease K at 37 °C for 30 min and then purified using phenol–chloroform. Finally, the ChIP DNA was used for library generation using the QIAseq Ultralow Input Library Kit (QIAGEN) following the manufacturer’s protocol. For ChIP-PCR, the ChIP DNA was used for the qPCR assay. The sequences of all primers are provided in the Supplementary Table.

#### ChIP-Seq analysis

Cutadapt (v2.5) was used to trim adapters and filter raw data to get clean data for next step. FastQC (v0.11.9) were used to perform the quality control of raw fastq data and clean fastq data. Next, Bowtie2 (v2.5.1) were chosen to perform genome alignment of clean data to the reference Homo sapiens genome (hg38). We executed the alignment result quality control based on the ENCODE4 Histone ChIP-seq Pipelines (https://www.encodeproject.org/pipelines/ENCPL809GEM/) and get the QC report for each sample. Peak calling were performed by using MACS2 (v2.1.2) with the parameters macs2 callpeak -t IP.bam -c input.bam -g hs -q 0.05 -m 5 50. After peak calling, R package ChIPQC were chosen to assess the data quality of already aligned or peak-called reads. Deeptools (v2.0) were chosen to transform indexed BAM file into bigwig file.

Next, ROSE (RANK ORDERING OF super-enhancers) algorithm (v1.3.1) were used to perform typical and super-enhancer calling with parameters: ROSE_main.py -g HG38 -i $(1).narrowPeak.bed -r $(1)_H3K27AC.bam -c $(2)_H3K27AC_INPUT.bam -o./$(1)/ -s 12500 -t 2500. Briefly, the H3K27ac ChIP-Seq peaks file identified by MACS2 and the H3K27ac ChIP-Seq BAM file were used as input for the algorithm, intergenic and intronic H3K27ac peaks within 12.5 kb were stitched together to define a single entity spanning a genomic region as enhancers. The stitched and individual enhancers without neighboring peaks within 12.5 kb were ranked by the level of H3K27ac signal in the genomic region. The stitched or individual enhancers with an H3K27ac intensity above a cutoff, where the slope of the distribution plot of H3K27ac ChIP-seq intensity is 1, were defined as SEs and the remaining enhancers were considered TEs. All enhancer regions are plotted in an increasing order based on their H3K27ac signal.

To evaluate the distribution characteristics and corresponding visualization of ChIP-Seq data, we used NGSplot, which is an R package. We aligned the genome using bowtie2 (v2.5.1) and sorted and indexed the BAM file of each cell line using samtools. We downloaded the Homo sapiens genome (hg38) from the Google driver file of NGSplot and then performed the metagene plot using NGSplot.r. The Input BAM file has removed background. The corresponding parameter is ngs.plot.r -G hg38 -c indexed.bam -R SE.bed -O SE_bed_3kb -L 3000. NGSplot can normalize the whole region of super-enhancer and divide them into intervals of unified standard. The signal of each bin in each interval was calculated and used to draw a continuous curve. The height of the curve represents the difference in H3K27ac signal within the specified region of MetR and WT cells. We used NGSplot to evaluate the average H3K27ac signal in the super-enhancer region in MetR cell lines and WT cell lines.

Homer software (v4.8) was applied to perform motif enrichment analysis with the parameters perl findMotifGenome.pl SuperEnhancers.bed hg38 homer_out/ -mcheck homer/data/knownTFs/vertebrates/all.motifs. The complete motif data source was obtained from Homer’s built-in motif data. Homer annotatePeaks.pl were applied to annotate the super-enhancer-associated genes and get peak density with the parameters perl annotatePeaks.pl super-enhancer.bed hg38 –bedGraph. DAVID (https://david.ncifcrf.gov/) and R package clusterProfiler (v4.6.0) was chosen to perform functional enrichment analysis of super-enhancer-associated genes. FIMO software were applied to perform motif scan of super-enhancer regions, according to the methods of a newly published study.^37^ Statistically significant motif matches identified by FIMO were defined as those with a *P* value < 0.05. The motif pwm matrix file were downloaded from JASPAR2022 database.

Public transcription factor ChIP-Seq data were downloaded from the Cistrome Data Browser (http://cistrome.org/db/#/) to verify the transcription factor predictions in our target super-enhancer region.

### RNA sequencing and data analysis

#### RNA libraries construction & sequencing

The total RNA of cells was isolated by using TRIzol and used for RNA sequencing. RNA quantification was performed with a Qubit 3.0 spectrophotometer (Thermo Fisher, MA, USA). Library preparation was performed by Epibiotek (Guangzhou, China). Briefly, total RNA was treated with the GeneRead™ rRNA Depletion Kit (Qiagen, Hilden, Germany, Cat No. 180211) to remove ribosomal RNA. rRNA-depleted RNA was fragmented and then used to construct strand-specific RNA libraries by using the VAHTS Stranded RNA-seq Library Prep Kit for Illumina (Vazyme, Nanjing, China, Cat. No NR602) according to the manufacturer’s instructions. Library quality was determined on a Qseq100 Bio-Fragment Analyzer (Bioptic, Taiwan, China). The strand-specific libraries were sequenced.

### Data analysis of RNA Sequencing

Adaptor and primer sequences from the library were trimmed. Following trimming, sequence reads were then aligned to the homo sapiens genome (version Hg38) using Hisat2 followed by a post-alignment quality check to assess the performance of the alignment. After alignment, HTseq were used to calculate the counts of the Reads mapped the genome. FPKM (fragments per kilobase million reads) was used to standardize the expression data, which allowed the comparison of gene expression levels between each group. We applied DESeq2 algorithm to detect the differentially expressed genes (DEGs) with the following criteria: (i) |log2FC | >1; (ii) FDR < 0.05. Volcano Plots were drawn by the R based on the differential expression analysis, and the color was determined by the filtering criteria. Gene ontology (GO) and pathway enrichment analysis were performed by DAVID online tools (https://david.ncifcrf.gov/) and “clusterProfiler” package. The analysis of the DAVID online tools was conducted on two independent gene lists containing 602 upregulated genes (log2FC ≥ 1, FDR < 0.05) and 687 downregulated genes (log2FC ≤ −1, FDR < 0.05) in DU145-MetR vs. DU145-WT group and 996 upregulated genes (log2FC ≥ 1, FDR < 0.05) and 406 downregulated genes (log2FC ≤ −1, FDR < 0.05) in 22RV1-MetR vs. 22RV1-WT group. The DEGs were further fitted into pathway enrichment analysis by “clusterProfiler” package using the annotation of “KEGG”. Adjust *P* value < 0.05 was considered statistically significant enrichment. Circle plot was performed to visualize the linkages of DEGs and enriched concepts. Cell cycle score of each patient in TCGA-PRAD was evaluated by gene set enrichment analysis using the annotation of “cell cycle” gene set from “KEGG”. Then, Pearson correlation analysis was conducted to examine the relation between four candidate genes (PTGR1, CEBPD, DDIT4, and EEF1A1) and cell cycle.

### Single-cell RNA-Seq and bioinformatics analysis

The single-cell RNA-Seq process mainly includes four steps: single-cell isolation, whole-genome amplification, high-throughput sequencing, and data analysis. The preresistant DU145 cell model and wild-type DU145 cells were collected by centrifugation at 300×*g* for 5 min, and then single cells were immediately isolated by using 10x Genomics technology. After reverse transcription, the constructed cDNA library was used for RNA-Seq. Sequencing was performed by using the GPL27804 (Homo sapiens) platform and the Illumina NovaSeq 6000 System (Illumina, USA). Cell Ranger (version 2.2.0) was used to process the raw data, demultiplex cellular barcodes, map the reads to the transcriptome, and down sampled reads. These processes produced a raw unique molecular identifier (UMI) count matrix, which was leveraged to create the Seurat object by using the R package “Seurat” (version 4.0.1). Cells with a UMI number <500, with over 20% mitochondrial-derived UMI counts or with fewer than 250 genes detected were considered low-quality cells and were filtered out. Finally, 15285 single cells were retained for subsequent analysis. After quality control, the UMI count matrix was normalized to the total expression in the corresponding cell, multiplied by a scaling factor of 10000, and then log2-transformed. To adjust for batch effects between samples generated by technical and biological sources, we performed the standard anchor-based preprocessing procedure for removing potential batch effects. In this procedure, the top 5000 variable features were used to identify the potential anchors by the “FindIntegrationAnchors” function in Seurat. Then, the data were integrated by the “IntegrateData” function. To reduce the dimensionality of the scRNA-Seq dataset, principal component analysis (PCA) was performed on the integrated data matrix. By the Elbowplot function of Seurat, the majority of the variance was captured in the first 20 PCs, which were utilized to perform the downstream analysis. The main cell clusters were identified with the “FindClusters” function offered by Seurat with the resolution set as 0.2. The distribution of cells and clustering performance were visualized with 2D t-SNE plots. Differentially expressed genes in each cluster were identified based on the Wilcoxon rank-sum test, which was implemented in the Seurat “Findmarker” function.

### Dual-luciferase reporter assay

Both plasmids were constructed and purchased from Dongze Biotech Co., Ltd. (Guangzhou, China). The dual-luciferase reporter assay was performed by using the Dual-Luciferase® Reporter Assay System. Cells were seeded into each well of 96-well plates for preparation. The supernatant was removed, and then 35 μl of PBS and 35 μl of d-Luciferin were added. After mixing for 10 min, the fluorescence value was determined. Finally, the fluorescence value was determined again after adding 35 μl of Stop reagent and mixing for 10 min.

### Cell cycle and apoptosis assays

The cell cycle assay was performed by using a Cell Cycle Staining Kit (MULTI SCIENCE, China). A total of 1 × 10^6^ cells from the control and experimental groups were transferred to a 1.5-ml centrifuge tube. The supernatant was removed, and the cell pellet was collected after centrifugation at 1800 rpm for 3 min. The cell pellet was mixed with 1 ml DNA staining solution and 10 μl permeabilization solution and then incubated at 37 °C in the dark for 30 min. For the apoptosis assay, the Annexin V-FITC/PI Apoptosis Detection Kit (70-AP101–100) was purchased from Multi Sciences, China and used according to the instructions. The stained cells were analyzed via flow cytometry on a BD FACSVerse instrument (BD Biosciences, USA). FlowJo and ModFit software were used to analyze the data.

### Cell proliferation assays

Cell proliferation was tested by a colony formation assay and a Cell Counting Kit-8 (CCK-8) assay. The CCK-8 kit was purchased from Meilunbio Co., Ltd. China (MA0218). The assay was performed according to a previously described protocol.^53^

### Cell migration and invasion assays

Cell migration and invasion were evaluated by a wound-healing assay and a transwell invasion assay, as previously described.^53^ Matrigel matrix was purchased from Corning, USA (Cat. No: 354,234) and diluted to the working concentration a day before the experiment.

### qRT‒PCR assay

Total RNA in cells was obtained by using an RNeasy Mini Kit (Qiagen). mRNA expression was quantified by using qRT‒PCR according to the protocol of our previous studies.^53^ The sequences of all primers used for qPCR are provided in the Supplementary Table.

### Western blot analysis

The concentration of protein extracted from each cell line was obtained by using a BCA Protein Assay Kit (Thermo Fisher Scientific). The protein expression level of the target gene was quantified by using western blot analysis according to the protocol of our previous studies.^53^ The antibodies are described in the Supplementary Table.

### Immunohistochemistry

PTGR1 protein expression in tissues from the nude mouse subcutaneous xenograft model was evaluated by IHC in accordance with our previously published protocols.^53^ The antibodies are described in the Supplementary Table.

### Immunofluorescence

The immunofluorescence samples were prepared in accordance with our previously published protocols.^53^ The samples were imaged using a confocal laser scanning microscope (LSM880, Zeiss, Germany). The antibodies are described in the Supplementary Table.

### Statistical analysis and Bioinformatics

The version 21.0 SPSS for Windows (SPSS Inc, IL, USA) software and the R (version 4.2.2) were used for statistical analysis and visualization. The biochemical recurrence (BCR)-free survival was evaluated using the Kaplan–Meier method and the log-rank test based on the optimal cutoff values generated by the survminer R package. To explore the potentially biological alteration related to PTGR1 in prostate cancer, a differentially expressed analysis was first performed between the high- and low-expression subgroups based on the median value of PTGR1 expression in the entire cohort from TCGA. We then arranged the genes in descending order based on the magnitude of the absolute value of their Log2 FC. The ordered gene list was further fitted in the GSEA analysis by the R package, “clusterProfilter”. Adjusted *P* value < 0.05 were considered to be statistically significant. The process of the Bioinformatics analysis was in accordance with our previous publishment.^54^ Gene set variation analysis (GSVA) was performed to measure the cell cycle score for patients in TCGA-PRAD datasets with the annotation of “cell cycle” based on Kyoto encyclopedia of genes and genomes (KEGG). Pearson’s correlation analysis was leveraged to examine the relationship between cell cycle score and four marker genes of metformin resistance. Continuous variables were expressed as mean ± SD or mean ± SEM. Differences among groups were assessed using the Independent-Samples *t* test. Differences were considered statistically significant when the *P* value was less than 0.05.

### Supplementary information

Supplementary_Materials

Quality control reports of H3K27ac ChIP-Seq

Quality control reports of RUNX3/SRF ChIP-Seq

## Data Availability

The raw sequence data reported in this paper have been deposited in the Genome Sequence Archive (Genomics, Proteomics & Bioinformatics 2021) in the National Genomics Data Center (Nucleic Acids Res 2022), China National Center for Bioinformation/Beijing Institute of Genomics, Chinese Academy of Sciences (GSA-Human: HRA003867) that are publicly accessible at https://ngdc.cncb.ac.cn/gsa-human. The Cancer Genome Atlas (TCGA) data, including RNA-sequencing data and curated clinical phenotypes of prostate cancer, were derived from the University of California Santa Cruz (UCSC) Xena dataset (https://xenabrowser.net/). Data of Cambridge, CancerMap, and GSE107299 databases were obtained from the Gene Expression Omnibus (GEO, http://www.ncbi.nlm.nih.gov/geo/).
