# Supplementary material for: Metformin escape in prostate cancer by activating the PTGR1 transcriptional program through a novel super-enhancer
Source: Signal Transduct Target Ther. 2023 Aug 16;8:303. doi: 10.1038/s41392-023-01516-2 (PMC10427640; doi:10.1038/s41392-023-01516-2)
Supplement: Supplementary file 1 — Supplementary_Materials [file 41392_2023_1516_MOESM1_ESM.docx]

Supplementary Materials for

Metformin escape in prostate cancer by activating the PTGR1 transcriptional program through a novel super-enhancer

Authors: Jianheng Ye^1*^, Shanghua Cai^1,2,3*^, Yuanfa Feng^1,2*^, Jinchuang Li^1^, Zhiduan Cai^1^, Yulin Deng^2^, Ren Liu^1^, Xuejin Zhu^1^, Jianming Lu^1^, Yangjia Zhuo^1^, Yingke Liang^1^, Jianjiang Xie^1^, Yanqiong Zhang^4^, Huichan He^2^, Zhaodong Han^1^✉, Zhenyu Jia ^5,6^✉ and Weide Zhong^1,2,3,7^✉

Correspondence to: Weide Zhong. E-mail: zhongwd2009@live.cn,

Zhenyu Jia. Email: zhenyuj@ucr.edu and

Zhaodong Han. Email: 75028579@qq.com

**This PDF file includes:**

Materials and Methods

Supplementary Text

Supplementary Figures. S1 to S9

Supplementary Tables S1 to S3

**Other Supplementary Materials for this manuscript include the following:**

Quality control reports of H3K27ac ChIP-Seq

Quality control reports of RUNX3/SRF ChIP-Seq

Certificate of editing

Materials and Methods

Construction of metformin resistant prostate cancer cells

The prostate cancer cell lines DU145, PC3, and 22RV1 were used to construct metformin-resistant cell models. All human cancer cell lines were purchased from the American Type Culture Collection (ATCC) (Manassas, VA, USA) and cultured with their corresponding medium. We first calculated the half-maximal inhibitory concentration (IC_50_) of metformin in each cancer cell line at 72 h using the Cell Counting Kit-8 (CCK-8) assay. Then, 50,000 cancer cells were seeded in each well of 6-well plates and cultured with the IC_50_ of metformin for 72 h. After that, approximately 50% of the cells in each well were transferred to culture dishes and cultured with half of the IC_50_ of metformin for one month. To construct the preresistant PCa cell model, 50,000 DU145 cells were cultured with the IC_50_ of metformin for 72 h and then cultured with half of the IC_50_ of metformin for two weeks. Finally, the resistance phenotype of the cell models was verified by a CCK-8 assay and in subcutaneous xenograft tumor models.

Establishment of subcutaneous xenograft tumor model

Animal experiments were performed in compliance with the guidelines of the Animal Ethics Committee at South China University of Technology (Guangzhou, Guangdong, China). All BALB/c nude mice were divided into three groups: ① non-metformin feeding group; ② metformin feeding group (250 mg/kg metformin diluted in the drinking water); and ③ intermittent feeding group (metformin administration at intervals of 3 days). A total of 1×10^6^ cells from the control and MetR groups were injected into the left and right flank, respectively, of each BALB/c nude mouse. The tumor volume was calculated using the following formula: Volume (mm^3^) = width^2^ (mm^2^) × length (mm)/2.

Cell lines construction and transfection

DU145 and 22RV1 cells were infected with lentivirus containing the PTGR1 overexpression plasmid with a puro cassette and GFP tag. Stable cell lines were selected via growth in medium containing 3 g/mL puromycin 2 days after transfection. The targeting siRNA and negative control siRNA and the transfection reagent were obtained from GenePharma. The siRNA sequences are provided in the Supplementary. The transfection efficacy was tested by Western blot analysis 72 hours after transfection. Both constructed cell lines were confirmed by Western blot analysis. The sequences are presented in the Supplementary.

Seahorse assay

A total of 8,000 DU145 cells and 16,000 22RV1 cells were seeded in each well of an XFe24 cell culture microplate and treated with medium with or without metformin for one day before the assay was performed. Mitochondrial function was determined by measuring the oxygen consumption rate using an XF Cell Mito Stress Test Kit (Agilent Technologies) according to the protocol of our previous study31. Glycolytic activity was determined by measuring the extracellular acidification rate using an XF Glycolysis Stress Test Kit. The results were analyzed using Wave 2.6.0 software (Seahorse Bioscience). The Seahorse XF Cell Mito Stress Test Kit and XF Glycolysis Stress Test Kit were purchased from Agilent Technologies.

ChIP-Seq and analysis

ChIP-Seq sample preparation & sequencing

H3K27ac, RUNX3 and SRF ChIP-Seq were performed using the EpiTM chromatin immunoprecipitation kit (Epibiotek, cat. no. R1802). First, a total of 2×10^6^ cells were collected and subjected to crosslinking with one percent formaldehyde for 10 min, and the reaction was then quenched with 0.125 M glycine for 5 min. To isolate nuclei, 1 mL lysis buffer was added, and the cell debris was collected by centrifugation at 2,400 × g and 4 °C for 10 min. After that, nuclei were located in the supernatant and subjected to enzymatic shearing to generate chromatin fragments of an average length of between 200 bp and 500 bp by incubation at 37 °C for 10 min. The supernatant was collected by centrifugation at 18,000 × g and 4 °C for 10 min. The supernatant was mixed with the ChIP reaction mix (protein A/G magnetic beads, ChIP IP buffer, antibody, protease inhibitor cocktail) and incubated with rotation at 4 °C overnight. On the second day, after washing and removing the protein A/G magnetic beads from the mixture, the chromatin was eluted in reverse cross-linking buffer at 65 °C for 3 h. Next, the ChIP DNA was mixed with RNase A and protease K at 37 °C for 30 min and then purified using phenol–chloroform. Finally, the ChIP DNA was used for library generation using the QIAseq Ultralow Input Library Kit (QIAGEN) following the manufacturer’s protocol. For ChIP-PCR, the ChIP DNA was used for the qPCR assay. The sequences of all primers are provided in the Supplementary table.

ChIP-Seq analysis

Cutadapt (v2.5) was used to trim adapters and filter raw data to get clean data for next step. FastQC (v0.11.9) were used to perform the quality control of raw fastq data and clean fastq data. Next, Bowtie2 (v2.5.1) were chosen to perform genome alignment of clean data to the reference Homo sapiens genome (hg38). We executed the alignment result quality control based on the ENCODE4 Histone ChIP-seq Pipelines (https://www.encodeproject.org/pipelines/ENCPL809GEM/) and get the QC report for each sample. Peak calling were performed by using MACS2 (v2.1.2) with the parameters macs2 callpeak -t IP.bam -c input.bam -g hs -q 0.05 -m 5 50. After peak calling, R package ChIPQC were chosen to assess the data quality of already aligned or peak called reads. Deeptools (v2.0) were chosen to transform indexed BAM file into bigwig file.

Next, ROSE (RANK ORDERING OF super-enhancers) algorithm (v1.3.1) were used to perform typical and super-enhancer calling with parameters: ROSE_main.py -g HG38 -i $(1).narrowPeak.bed -r $(1)_H3K27AC.bam -c $(2)_H3K27AC_INPUT.bam -o ./$(1)/ -s 12500 -t 2500. Briefly, the H3K27ac ChIP-Seq peaks file identified by MACS2 and the H3K27ac ChIP-Seq BAM file were used as input for the algorithm, intergenic and intronic H3K27ac peaks within 12.5 kb were stitched together to define a single entity spanning a genomic region as enhancers. The stitched and individual enhancers without neighboring peaks within 12.5 kb were ranked by the level of H3K27ac signal in the genomic region. The stitched or individual enhancers with an H3K27ac intensity above a cutoff, where the slope of the distribution plot of H3K27ac ChIP-seq intensity is 1, were defined as SEs and the remaining enhancers were considered TEs. All enhancer regions are plotted in an increasing order based on their H3K27ac signal.

To evaluate the distribution characteristics and corresponding visualization of ChIP Seq data, we used NGSplot, which is an R package. We aligned the genome using bowtie2 (v2.5.1) and sorted and indexed the BAM file of each cell line using samtools. We downloaded the Homo sapiens genome (hg38) from the Google driver file of NGSplot and then performed the metagene plot using NGSplot.r. The Input BAM file has removed background. The corresponding parameter is ngs.plot.r -G hg38 -c indexed.bam -R SE.bed -O SE_bed_3kb -L 3000. NGSplot can normalize the whole region of super-enhancer and divide them into intervals of unified standard. The signal of each bin in each interval was calculated and used to draw a continuous curve. The height of the curve represents the difference in H3K27ac signal within the specified region of MetR and WT cells. We used NGSplot to evaluate the average H3K27ac signal in the super-enhancer region in MetR cell lines and WT cell lines.

Homer software (v4.8) was applied to perform motif enrichment analysis with the parameters perl findMotifGenome.pl SuperEnhancers.bed hg38 homer_out/ -mcheck homer/data/knownTFs/vertebrates/all.motifs. The complete motif data source was obtained from Homer's built-in motif data. Homer annotatePeaks.pl were applied to annotate the super-enhancer-associated genes and get peak density with the parameters perl annotatePeaks.pl super-enhancer.bed hg38 –bedGraph. DAVID (https://david.ncifcrf.gov/) and R package clusterProfiler (v4.6.0) was chosen to perform functional enrichment analysis of super-enhancer-associated genes. FIMO software were applied to perform motif scan of super-enhancer regions, according to the methods of a newly published study38. Statistically significant motif matches identified by FIMO were defined as those with a p value < 0.05. The motif pwm matrix file was downloaded from JASPAR 2022 database.

Public transcription factor ChIP-Seq data were downloaded from the Cistrome Data Browser (http://cistrome.org/db/#/) to verify the transcription factor predictions in our target super-enhancer region.

RNA Sequencing and Data analysis

RNA libraries construction & sequencing

Total RNA of cells was isolated by using TRIzol and used for RNA sequencing. RNA quantification was performed with a Qubit 3.0 spectrophotometer (Thermo Fisher, MA, USA). Library preparation was performed by Epibiotek (Guangzhou, China). Briefly, total RNA was treated with the GeneRead™ rRNA Depletion Kit (Qiagen, Hilden, Germany, Cat No. 180211) to remove ribosomal RNA. rRNA-depleted RNA was fragmented and then used to construct strand-specific RNA libraries by using the VAHTS Stranded RNA-seq Library Prep Kit for Illumina (Vazyme, Nanjing, China, Cat. No NR602) according to the manufacturer’s instructions. Library quality was determined on a Qseq100 Bio-Fragment Analyzer (Bioptic, Taiwan, China). The strand-specific libraries were sequenced.

Data analysis of RNA Sequencing

Adaptor and primer sequences from the library were trimmed. Following trimming, sequence reads were then aligned to the homo sapiens genome (version Hg38) using Hisat2 followed by a post-alignment quality check to assess the performance of the alignment. After alignment, HTseq were used to calculate the counts of the Reads mapped the genome. FPKM（Fragments Per Kilo base Million Reads）was used to standardize the expression data, which allowed the comparison of gene expression levels between each group. We applied DESeq2 algorithm to detect the differentially expressed genes (DEGs) with the following criteria: i) |log2FC| > 1; ii) FDR < 0.05. Volcano Plots were drawn by the R based on the differential expression analysis and the color was determined by the filtering criteria. Gene ontology (GO) and pathway enrichment analysis were performed by DAVID online tools (https://david.ncifcrf.gov/) and "clusterProfiler" package. The analysis of the DAVID online tools was conducted on two independent gene lists containing 602 up-regulated genes (log2FC≥ 1, FDR < 0.05) and 687 down-regulated genes (log2FC≤ -1, FDR < 0.05) in DU145-MetR vs. DU145-WT group and 996 up-regulated genes (log2FC≥ 1, FDR < 0.05) and 406 down-regulated genes (log2FC≤ -1, FDR < 0.05) in 22RV1-MetR vs. 22RV1-WT group. The DEGs were further fitted into pathway enrichment analysis by "clusterProfiler" package using the annotation of "KEGG". Adjust p value <0.05 was considered statistically significant enrichment. Circle plot was performed to visualize the linkages of DEGs and enriched concepts. Cell cycle score of each patient in TCGA-PRAD was evaluated by gene set enrichment analysis using the annotation of "cell cycle" gene set from "KEGG". Then, pearson correlation analysis was conducted to examine the relation between four candidate genes (PTGR1, CEBPD, DDIT4, and EEF1A1) and cell cycle.

Single-cell RNA-Seq and bioinformatics analysis

The single-cell RNA-Seq process mainly includes four steps: single-cell isolation, whole-genome amplification, high-throughput sequencing and data analysis. The preresistant DU145 cell model and wild-type DU145 cells were collected by centrifugation at 300 × g for 5 min, and then single cells were immediately isolated by using 10x Genomics technology. After reverse transcription, the constructed cDNA library was used for RNA-Seq. Sequencing was performed by using the GPL27804 (Homo sapiens) platform and the Illumina NovaSeq 6000 System (Illumina, USA). Cell Ranger (version 2.2.0) was used to process the raw data, demultiplex cellular barcodes, map the reads to the transcriptome, and down sampled reads. These processes produced a raw unique molecular identifier (UMI) count matrix, which was leveraged to create the Seurat object by using the R package “Seurat” (version 4.0.1). Cells with a UMI number <500, with over 20% mitochondrial-derived UMI counts or with fewer than 250 genes detected were considered low-quality cells and were filtered out. Finally, 15,285 single cells were retained for subsequent analysis. After quality control, the UMI count matrix was normalized to the total expression in the corresponding cell, multiplied by a scaling factor of 10,000, and then log2-transformed. To adjust for batch effects between samples generated by technical and biological sources, we performed the standard anchor-based preprocessing procedure for removing potential batch effects. In this procedure, the top 5,000 variable features were used to identify the potential anchors by the “FindIntegrationAnchors” function in Seurat. Then, the data were integrated by the “IntegrateData” function. To reduce the dimensionality of the scRNA-Seq dataset, principal component analysis (PCA) was performed on the integrated data matrix. By the Elbowplot function of Seurat, the majority of the variance was captured in the first 20 PCs, which were utilized to perform the downstream analysis. The main cell clusters were identified with the “FindClusters” function offered by Seurat with the resolution set as 0.2. The distribution of cells and clustering performance were visualized with 2D tSNE plots. Differentially expressed genes in each cluster were identified based on the Wilcoxon rank-sum test, which was implemented in the Seurat “Findmarker” function.

Dual-luciferase reporter assay

Both plasmids were constructed and purchased from Dongze Biotech Co., Ltd. (Guangzhou, China). The dual-luciferase reporter assay was performed by using the Dual-Luciferase® Reporter Assay System. Cells were seeded into each well of 96-well plates for preparation. The supernatant was removed, and then 35 μl of PBS and 35 μl of D-Luciferin were added. After mixing for 10 min, the fluorescence value was determined. Finally, the fluorescence value was determined again after adding 35 μl of Stop reagent and mixing for 10 min.

Cell cycle and apoptosis assays

The cell cycle assay was performed by using a Cell Cycle Staining Kit (MULTI SCIENCE, China). A total of 1×10^6^ cells from the control and experimental groups were transferred to a 1.5 ml centrifuge tube. The supernatant was removed, and the cell pellet was collected after centrifugation at 1,800 rpm for 3 min. The cell pellet was mixed with 1 ml DNA staining solution and 10 μl permeabilization solution and then incubated at 37 °C in the dark for 30 min. For the apoptosis assay, the Annexin V-FITC/PI Apoptosis Detection Kit (70-AP101–100) was purchased from Multi Sciences, China and used according to the instructions. The stained cells were analyzed via flow cytometry on a BD FACSVerse instrument (BD Biosciences, USA). FlowJo and ModFit software were used to analyze the data.

Cell proliferation assays

Cell proliferation was tested by a colony formation assay and a Cell Counting Kit-8 (CCK-8) assay. The CCK-8 kit was purchased from Meilunbio Co., Ltd. China (MA0218). The assay was performed according to a previously described protocol.

Cell migration and invasion assays

Cell migration and invasion were evaluated by a wound healing assay and a transwell invasion assay as previously described. Matrigel matrix was purchased from Corning, USA (Cat. No: 354,234) and diluted to the working concentration a day before the experiment.

qRT‒PCR assay

Total RNA in cells was obtained by using a RNeasy Mini Kit (Qiagen). mRNA expression was quantified by using qRT‒PCR according to the protocol of our previous studies. The sequences of all primers used for qPCR are provided in the Supplementary table.

Western blot analysis

The concentration of protein extracted from each cell line was obtained by using a BCA Protein Assay Kit (Thermo Fisher Scientific). The protein expression level of the target gene was quantified by using Western blot analysis according to the protocol of our previous studies. The antibodies are described in the Supplementary table.

Immunohistochemistry

PTGR1 protein expression in tissues from the nude mouse subcutaneous xenograft model was evaluated by IHC in accordance with our previously published protocols. The antibodies are described in the Supplementary table.

Immunofluorescence

The immunofluorescence samples were prepared in accordance with our previously published protocols. The samples were imaged using a confocal laser scanning microscope (LSM880, Zeiss, Germany). The antibodies are described in the Supplementary table.

Statistical analysis and Bioinformatics

The version 21.0 SPSS for Windows (SPSS Inc, IL, USA) software and the R (version 4.2.2) were used for statistical analysis and visualization. The biochemical recurrence (BCR)-free survival was evaluated using the Kaplan-Meier method and the log-rank test based on the optimal cut-off values generated by the survminer R package. To explore the potentially biological alteration related to PTGR1 in prostate cancer, a differentially expressed analysis was first performed between the high- and low-expression subgroups based on the median value of PTGR1 expression in the entire cohort from TCGA. We then arranged the genes in descending order based on the magnitude of the absolute value of their Log2 FC. The ordered gene list was further fitted in the GSEA analysis by the R package, ‘clusterProfilter’. Adjusted p value <0.05 were considered to be statistically significant. The process of the Bioinformatics analysis was in accordance with our previous publishment. Gene set variation analysis (GSVA) was performed to measure the cell cycle score for patients in TCGA-PRAD datasets with the annotation of “cell cycle” based on Kyoto encyclopedia of genes and genomes (KEGG). Pearson’s correlation analysis was leveraged to examined the relationship between cell cycle score and four marker genes of metformin resistance. Continuous variables were expressed as mean±SD or mean±SEM. Differences among groups were assessed using the Independent-Samples t test. Differences were considered statistically significant when the P value was less than 0.05.

Supplementary Text

Subhead

Type or paste text here. This should be additional explanatory text, such as: extended technical descriptions of results, full details of mathematical models, extended lists of acknowledgments, etc. It should not be additional discussion, analysis, interpretation, or critique.

<insert page break then Fig. S1 here>


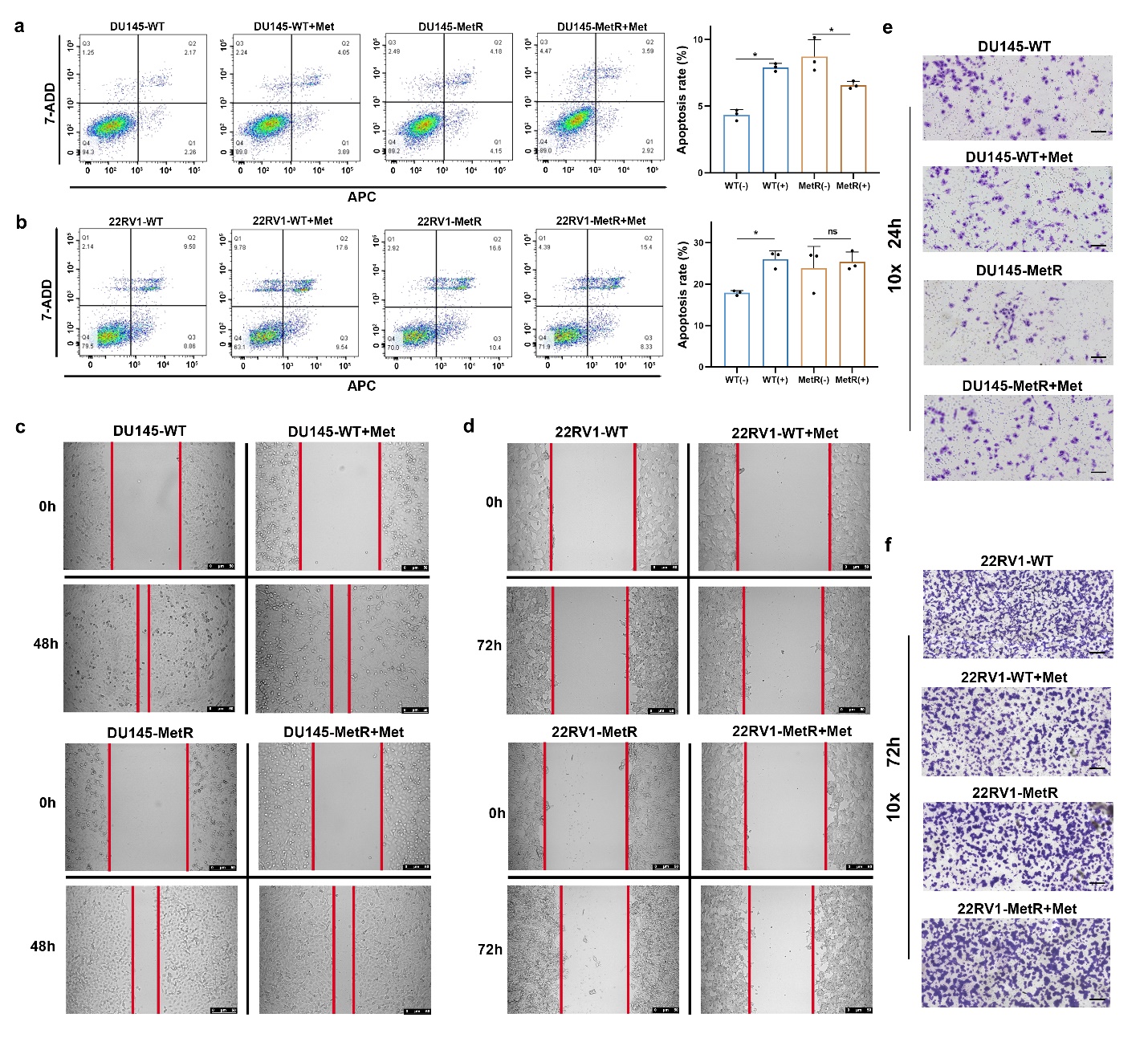


Supplementary Figure. S1.

Supplementary Figure S1. Metformin exerts similar influence on cell apoptosis, migration and invasion in metformin resistant PCa cells and wild type cells. a: The apoptosis rate of each cell group in DU145 cells was analyzed by flow cytometric analysis. 20mM of metformin was used in the treatment group. b: The apoptosis rate of each cell group in 22RV1 cells was analyzed by flow cytometric analysis. 10mM of metformin was used in the treatment group. c: Cell migration of each cell group in DU145 cells was detected by wound healing assay. 20mM of metformin was used in the treatment group. d: Cell migration of each cell group in 22RV1 cells was detected by wound healing assay. 10mM of metformin was used in the treatment group. e: Cell invasion of each cell group in DU145 cells was detected by transwell invasion assay. 20mM of metformin was used in the treatment group. f: Cell invasion of each cell group in 22RV1 cells was detected by transwell invasion assay. 10mM of metformin was used in the treatment group.

<insert page break then Fig. S2 here>


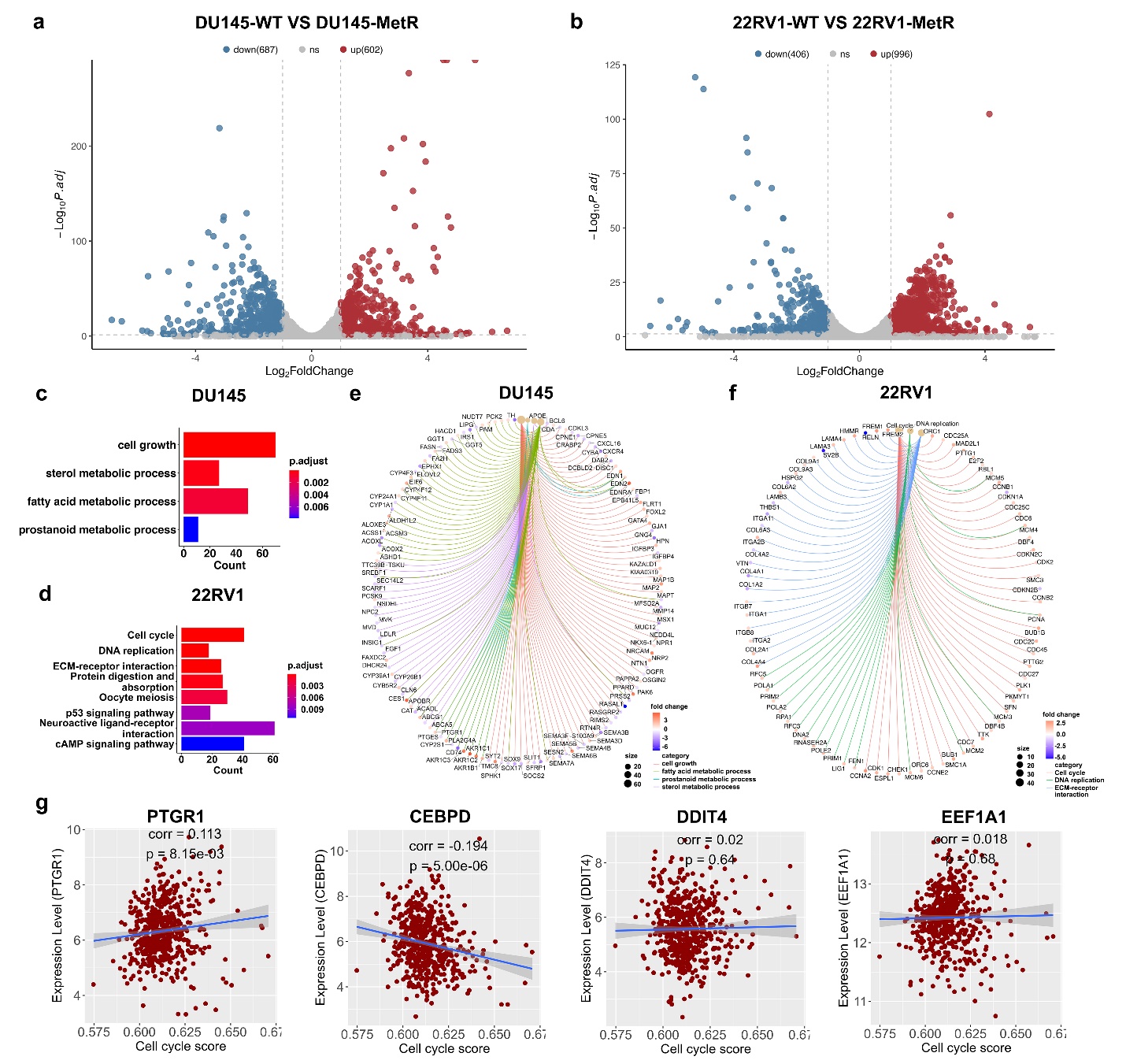


Supplementary Figure. S2.

Supplementary Figure S2. RNA-Seq analysis of DU145-WT cells and DU145-MetR cells, 22RV1-WT cells and 22RV1-MetR cells. a: Volcano plot for the differentially expressed genes between DU145-WT group and DU145-MetR group. Red dots represented up-regulated genes (log2Fold change>1 and P value <0.05) and blue dots represented down-regulated genes (log2Fold change <−1 and P value <0.05). b: Volcano plot for the differentially expressed genes between 22RV1-WT group and 22RV1-MetR group. Red dots represented up-regulated genes (log2Fold change>1 and P value <0.05) and blue dots represented down-regulated genes (log2Fold change <−1 and P value <0.05). c: Pathway analysis using the gene set of differentially expressed genes between DU145-WT group and DU145-MetR group. d: Pathway analysis using the gene set of differentially expressed genes between 22RV1-WT group and 22RV1-MetR group. e-f: The list of metabolic DEGs and cell cycle-related DEGs. g: Correlation between mRNA expression of four marker genes of metformin resistance cluster and the expression of cell cycle related genes in the TCGA-PRAD (TCGA, The Cancer Genome Atlas; PRAD, prostate adenocarcinoma) database.

<insert page break then Fig. S3 here>


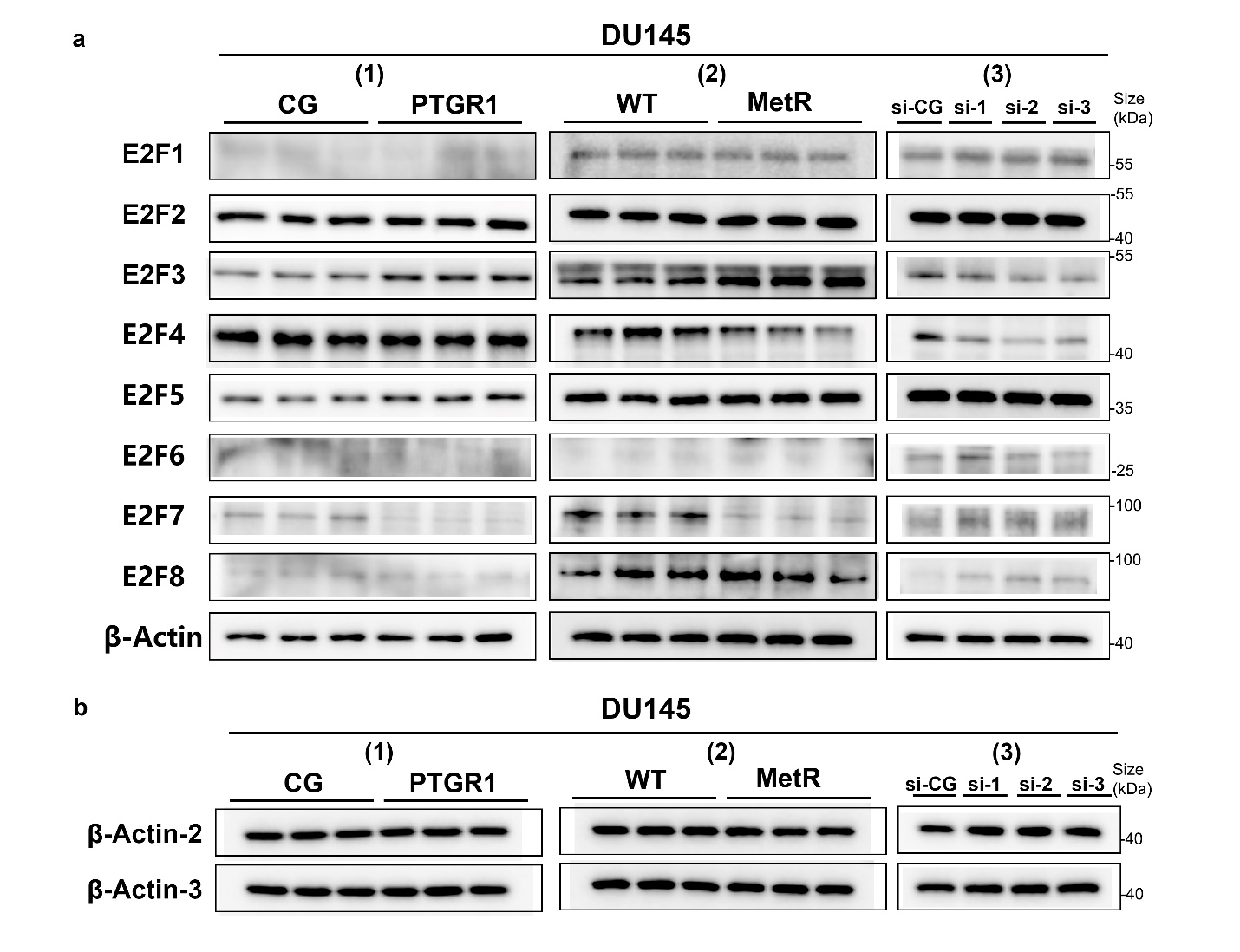


Supplementary Figure. S3.

Supplementary Figure S3. The relations of PTGR1 and E2F transcription factor family markers were analyzed by Western blot analyze. a: (1) Relative expression of E2F transcriptional factor family markers in DU145 cells (control group, CG) versus DU145 cells with PTGR1 up-regulation (PTGR1). (2) Relative expression of E2F transcriptional factor family markers in DU145-wild type cells (WT) versus DU145 cells with metformin resistance (MetR). (3) Relative expression of E2F transcriptional factor family markers in DU145 cells (control group, si-CG) versus DU145 cells with PTGR1 down-regulation (si-1, 2, 3). b: Additional beta actin bands of samples used in figure S3a.

<insert page break here>


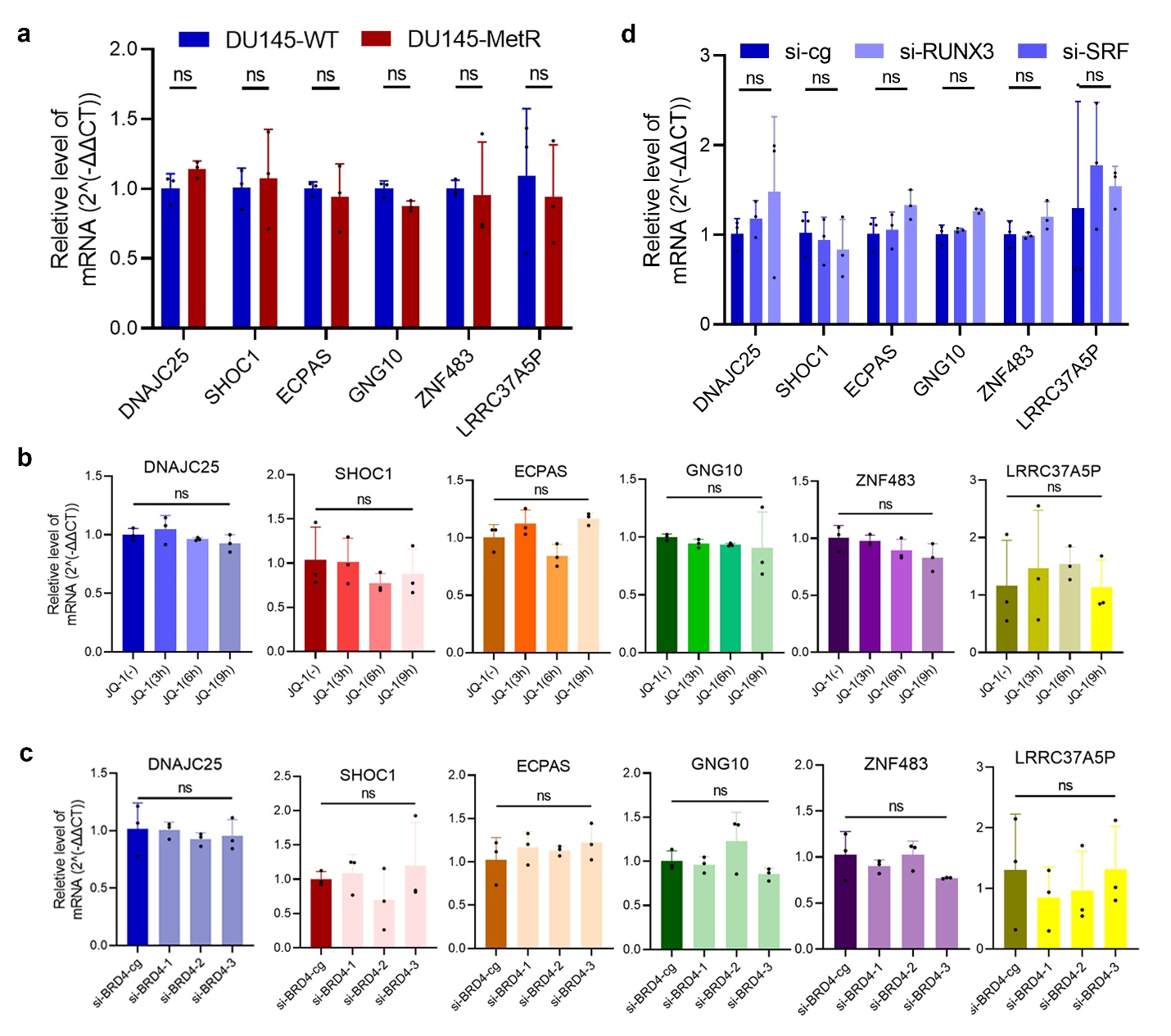


Supplementary Figure. S4.

Supplementary Figure S4. Expression levels of 6 neighboring genes, including DNAJC2, GNG10, ECPAS, LRRC37A5P, SHOC1, ZNF48, were analyzed by qRT-PCR assay. a: mRNA expression levels of 6 neighboring genes in DU145-MetR cells were measured by qRT-PCR assay. (n=3) b: mRNA expression levels of 6 neighboring genes were measured by qRT-PCR assay in DU145-MetR cells with JQ1 treatment for 3h, 6h and 9h respectively. (n=3) c: mRNA expression levels of 6 neighboring genes were measured by qRT-PCR assay in the condition of BRD4 down-regulated by siRNA. (n=3) d: mRNA expression levels of 6 neighboring genes were measured by qRT-PCR assay in DU145-MetR cells with SRF or RUNX3 down-regulated. (n=3) (ns, p>0.05)

<insert page break here>


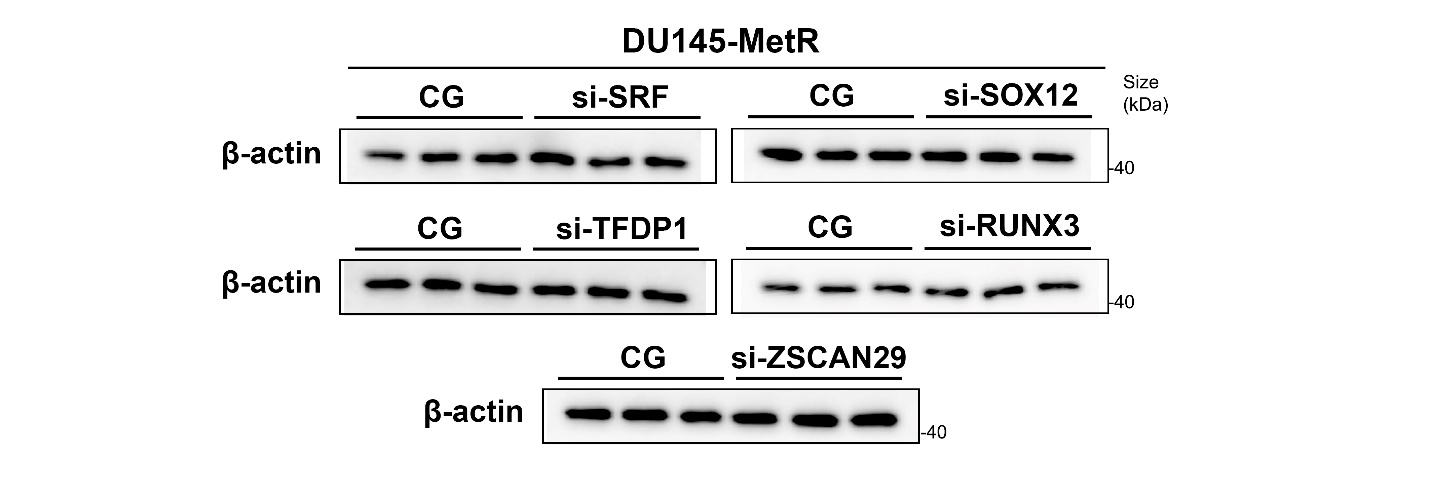


Supplementary Figure. S5.

Supplementary Figure S5. The beta-actin protein bands of the samples used in figure 6g.

<insert page break here>


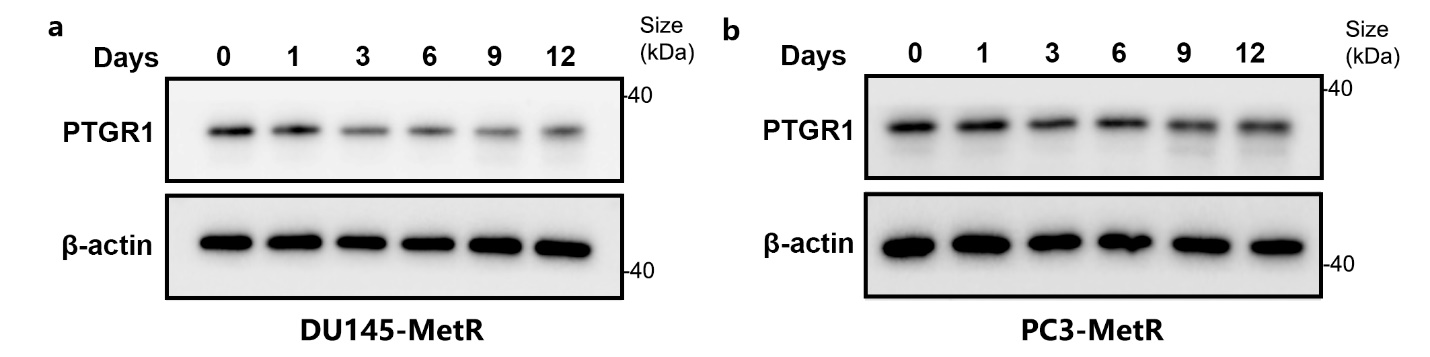


Supplementary Figure. S6.

Supplementary Figure S6. The expression of PTGR1 in MetR cells was decreased by metformin withdrawal in a time dependent manner. a: Western blot analysis were used to evaluate the expression of PTGR1 in DU145-MetR cells with metformin withdrawal for 0, 1, 3, 6, 9, 12 days. b: Western blot analysis were used to evaluate the expression of PTGR1 in PC3-MetR cells with metformin withdrawal for 0, 1, 3, 6, 9, 12 days.

<insert page break here>


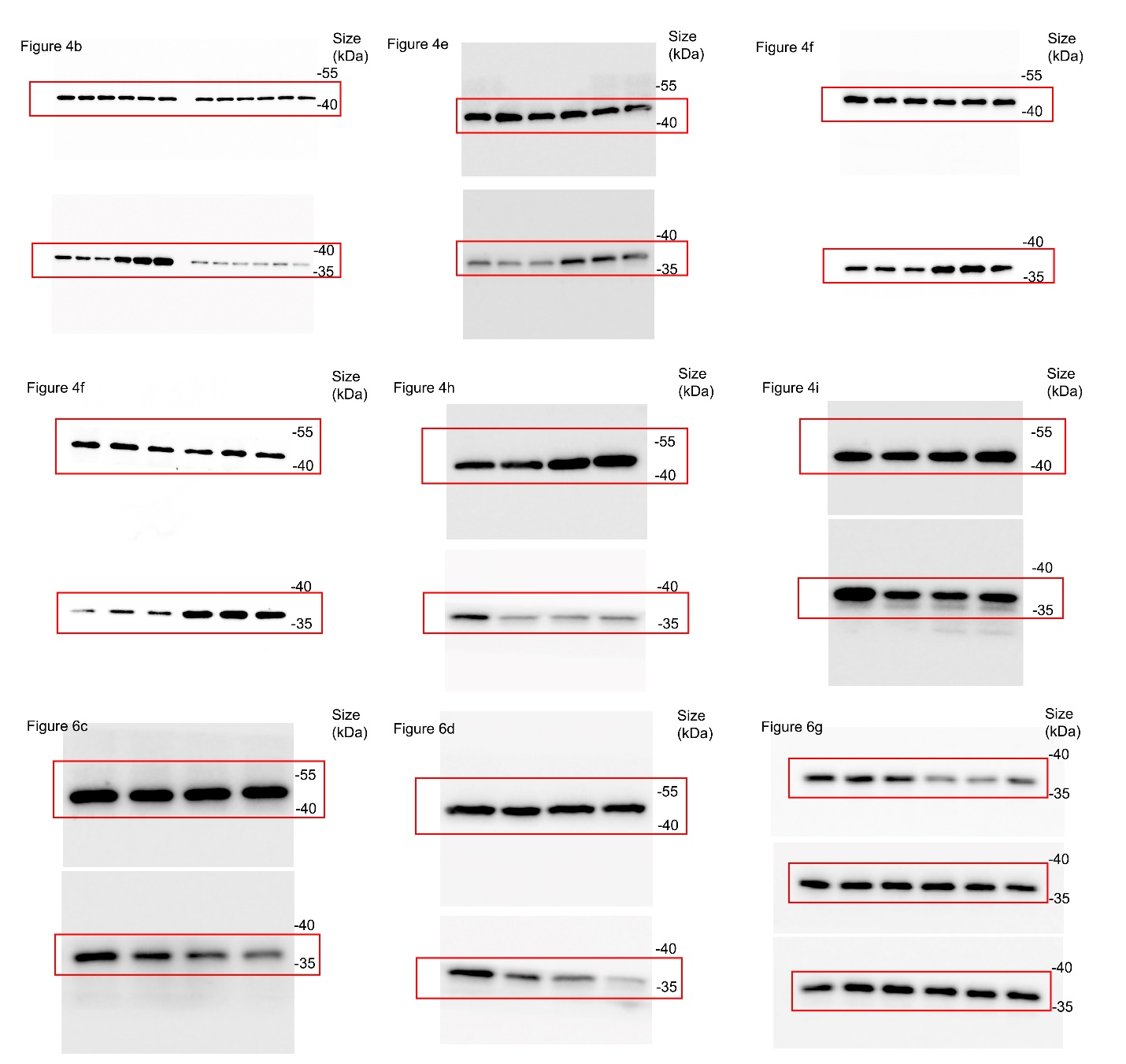


Supplementary Figure. S7.

Supplementary Figure S7-9 are the raw data of Western blot and gating strategies of apoptosis analysis.

<insert page break here>


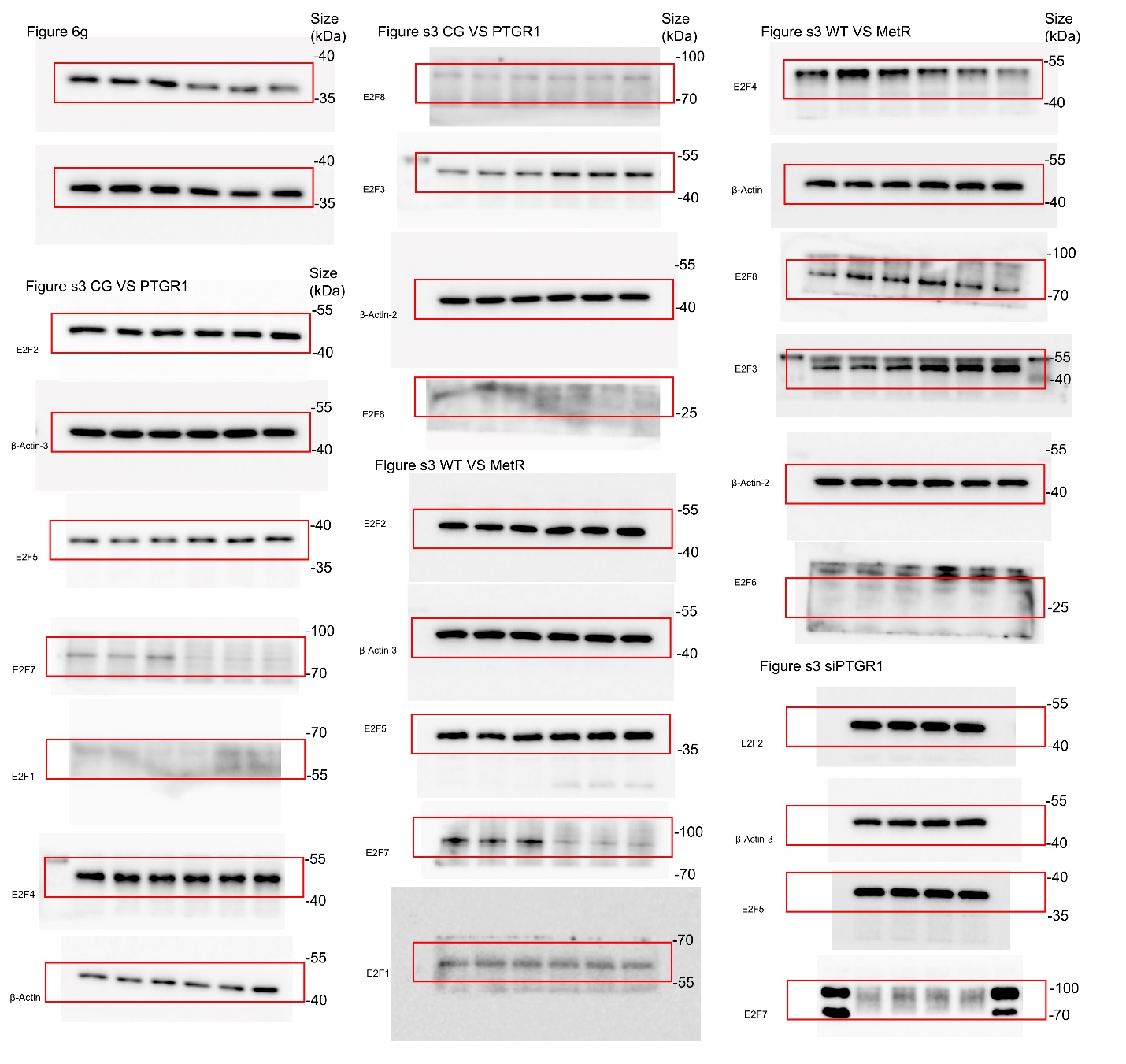


Supplementary Figure. S8.

Supplementary Figure S7-9 are the raw data of Western blot and gating strategies of apoptosis analysis.

<insert page break here>


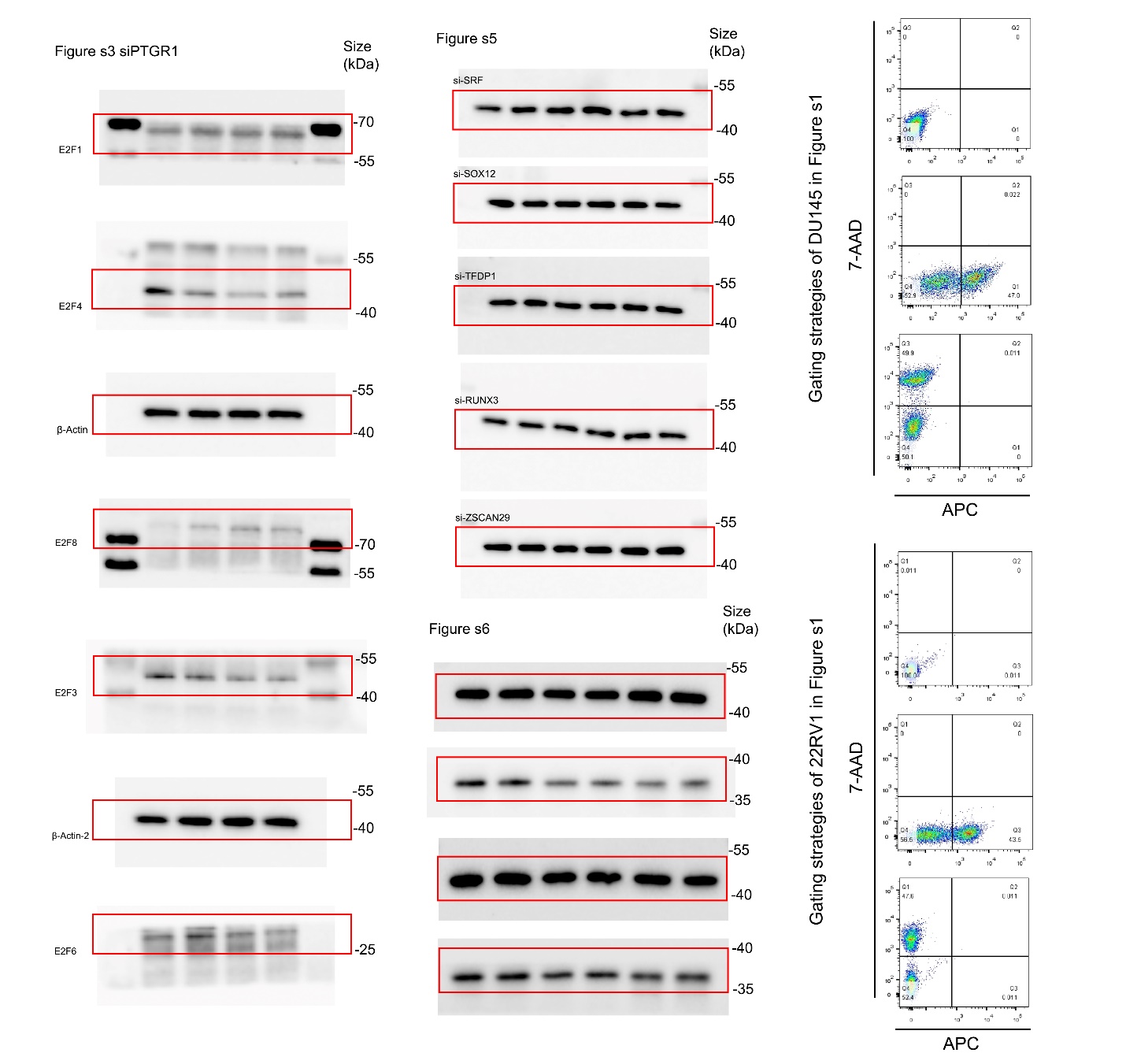


Supplementary Figure. S9.

Supplementary Figure S7-9 are the raw data of Western blot and gating strategies of apoptosis analysis.

Supplementary Table S1.

| Sequence of siRNA used in this study | | |
| --- | --- | --- |
| Name of target |  | 5'-3' |
| SRF（h）-si-1 | sense | CGGCGUUCACAGUCACCAA TT |
|  | antisense | UUGGUGACUGUGAACGCCG TT |
| SRF（h）-si-2 | sense | CCAGUGUCUGCUAGUGUCA TT |
|  | antisense | UGACACUAGCAGACACUGG TT |
| SRF（h）-si-3 | sense | GGGACUGUGCUGAAGAGUA TT |
|  | antisense | UACUCUUCAGCACAGUCCC TT |
|  |  |  |
| SOX12（h）-si-1 | sense | GGGCCUUUCUUGCGCUCUAUU TT |
|  | antisense | AAUAGAGCGCAAGAAAGGCCC TT |
| SOX12（h）-si-2 | sense | GCUGCUUCACAGGAUGAAA TT |
|  | antisense | UUUCAUCCUGUGAAGCAGC TT |
| SOX12（h）-si-3 | sense | CAGGUGCUUAUUCCUAAUA TT |
|  | antisense | UAUUAGGAAUAAGCACCUG TT |
|  |  |  |
| RUNX3（h）-si-1 | sense | GCGGCAAGAUGGGCGAGAA TT |
|  | antisense | UUCUCGCCCAUCUUGCCGC TT |
| RUNX3（h）-si-2 | sense | CCAUCACUGUGUUCACCAA TT |
|  | antisense | UUGGUGAACACAGUGAUGG TT |
| RUNX3（h）-si-3 | sense | GGCUCACUCAGCACCACAA TT |
|  | antisense | UUGUGGUGCUGAGUGAGCC TT |
|  |  |  |
| ZSCAN29（h）-si-1 | sense | GUCGGAGUGCACGACUCAUUA TT |
|  | antisense | UAAUGAGUCGUGCACUCCGAC TT |
| ZSCAN29（h）-si-2 | sense | ACCAGAACGCUCCUCGCAAUU TT |
|  | antisense | AAUUGCGAGGAGCGUUCUGGU TT |
| ZSCAN29（h）-si-3 | sense | CGGUAUCUUCAUCAGGGUAAA TT |
|  | antisense | UUUACCCUGAUGAAGAUACCG TT |
|  |  |  |
| TFDP1（h）-si-1 | sense | CACAUUUGAAAUCCACGAUGA TT |
|  | antisense | UCAUCGUGGAUUUCAAAUGUG TT |
| TFDP1（h）-si-2 | sense | CGGGAAGCGCAACAGGAAA TT |
|  | antisense | UUUCCUGUUGCGCUUCCCG TT |
| TFDP1（h）-si-3 | sense | CACCCCCAGCACUCACUUU TT |
|  | antisense | AAAGUGAGUGCUGGGGGUG TT |
|  |  |  |
| BRD4（h）-si-1 | sense | CCUGAUUACUAUAAGAUCA TT |
|  | antisense | UGAUCUUAUAGUAAUCAGG TT |
| BRD4（h）-si-2 | sense | AGAUUGAAAUCGACUUUGA TT |
|  | antisense | UCAAAGUCGAUUUCAAUCU TT |
| BRD4（h）-si-3 | sense | UGAGCACAAUCAAGUCUAA TT |
|  | antisense | UUAGACUUGAUUGUGCUCA TT |
|  |  |  |
| PTGR1-Homo-299 | sense | AAGCCAGUACAAUAGUUCCTT |
|  | antisense | GGAACUAUUGUACUGGCUUTT |
| PTGR1-Homo-531 | sense | GGCAGAUUGCAAAGCUCAATT |
|  | antisense | UUGAGCUUUGCAAUCUGCCTT |
| PTGR1-Homo-611 | sense | GGAUUUGAUGUCGUCUUUATT |
|  | antisense | UAAAGACGACAUCAAAUCCTT |

<insert Table S1 here followed by a page break >

Supplementary Table S2.

| Sequence of primer used in this study | | |
| --- | --- | --- |
| Name of target |  | 5'-3' |
| PTGR1 | Forward | GACAACGCACTCCATTTCTGA |
|  | Reverse | TGCTGCATTAACCATCACTGTT |
| DNAJC25 | Forward | CAGCGTGTGTGCTATTTCGG |
|  | Reverse | GGCTAGGTAGCTGATTGCCTTA |
| ECPAS | Forward | AACCGCATGCACCAAAACTC |
|  | Reverse | AAGCCGAGCACTATCCATCG |
| GNG10 | Forward | TGGTAGAGCAGCTCAAGTTGGA |
|  | Reverse | GGCATCCTTGCAGGCATTCTG |
| LRRC37A5P | Forward | CCTGCGTCCTGCTAGCTATT |
|  | Reverse | GGGACCATAGCTGCTGGTTT |
| SHOC1 | Forward | TTGCTTCGAATCCCTTCATGT |
|  | Reverse | ACAGCTGAGACTCTCGTCCA |
| ZNF483 | Forward | AACTCTGGCCTCGACTGAAC |
|  | Reverse | ACCACCTAAACCTCTGTCTGAA |
| β-Actin | Forward | AGCGAGCATCCCCCAAAGTT |
|  | Reverse | GGGCACGAAGGCTCATCATT |
| GAPDH promoter | Forward | CATGGGTGTGAACCATGAGA |
|  | Reverse | GTCTTCTGGGTGGCAGTGAT |
| PTGR1 promoter | RUNX3-F1 | GAACCAACCGTGGCATCTG |
|  | RUNX3-R1 | AGGGATCGGAGCCAGCA |
|  | SRF-F2 | GATGTCAGAAGTTCGAGACCAGC |
|  | SRF-R2 | CAAGGAGCTGGGACTACAGACG |

<insert Table S2 here followed by a page break >

Supplementary Table S3.

| Antibodies of all proteins | | |
| --- | --- | --- |
| Name | Source | Identifier |
| PTGR1 | Proteintech | 13374-1-AP |
| PTGR1 | Abcam | ab222818 |
| E2F1 | Proteintech | 66515-1-lg |
| E2F2 | Affinity | AF4100 |
| E2F3 | Santa Cruz | sc-56665 |
| E2F4 | Proteintech | 10923-1-AP |
| E2F5 | Affinity | AF5137 |
| E2F6 | Affinity | AF0152 |
| E2F7 | Proteintech | 24489-1-AP |
| E2F8 | Affinity | DF2591 |
| β-Actin | Proteintech | 81115-1-RR |
| β-Actin | Ray antibody | RM2001 |

<insert Table S2 here followed by a page break >

Supplementary file 1.

Quality control reports of H3K27ac ChIP-Seq.

Supplementary file 2.

Quality control reports of RUNX3/SRF ChIP-Seq.
