# Supplementary material for: Metformin escape in prostate cancer by activating the PTGR1 transcriptional program through a novel super-enhancer
Source: Signal Transduct Target Ther. 2023 Aug 16;8:303. doi: 10.1038/s41392-023-01516-2 (PMC10427640; doi:10.1038/s41392-023-01516-2)
Supplement: Supplementary file 2 — Quality control reports of H3K27ac ChIP-Seq [file 41392_2023_1516_MOESM2_ESM.pdf]

ChIPQC Report

Overview

This report was generated using **ChIPQC**

The report provides both general and ChIP-seq specific quality metrics and diagnostic graphics to allow for the quantitative assessment of ChIP-seq quality.

The report is split into three main sections:

- **QC Summary** - Overview of results.
- **QC Results** - Full QC results and figures.
- **QC files and versions** - Files and program versions used in QC

QC Summary

Table 1. Summary of ChIP-seq filtering and quality metrics.

ID	Tissue	Factor	Condition	Replicate	Reads	Dup%	ReadL	FragL	RelCC	SSD	RiP%
22RV1MetR_input	22RV1MetR	H3K27ac	22RV1MetR_input	2	4749225	19	150	0	0	15	0.94
22RV1MetR_IP	22RV1MetR	H3K27ac	22RV1MetR_IP	1	4170298	23	149	300	0.44	15	50
22RV1WT_input	22RV1WT	H3K27ac	22RV1WT_input	2	4610655	21	150	0	0	6.7	3.5
22RV1WT_IP	22RV1WT	H3K27ac	22RV1WT_IP	1	3978495	19	149	300	0.44	11	38
DU145MetR_input	DU145MetR	H3K27ac	DU145MetR_input	2	7519350	22	149	299	0.38	12	2.8
DU145MetR_IP	DU145MetR	H3K27ac	DU145MetR_IP	1	9104194	27	149	299	0.65	9	22
DU145WT_input	DU145WT	H3K27ac	DU145WT_input	2	7766357	22	149	299	0.37	15	4
DU145WT_IP	DU145WT	H3K27ac	DU145WT_IP	1	9726192	26	149	300	0.64	6.9	27

Table 1contains a summary of filtering and quality metrics generated by the ChIPQC package. Further information on these metrics, their associated figures and additional quality measures can be found within the related QC Results subsections.

A short description of **Table 1** metrics is provided below:

- **ID -** Unique sample ID.
- **Tissue/Factor/Condition** - Metadata associated to sample.
- **Replicate** - Number of replicate within sample group
- **Reads** - Number of sample reads within analysed chromosomes.
- **Dup%** - Percentage of MapQ filter passing reads marked as duplicates
- **FragLen** - Estimated fragment length by cross-coverage method
- **SSD** - SSD score (htSeqTools)
- **FragLenCC** - Cross-Coverage score at the fragment length
- **RelativeCC** - Cross-coverage score at the fragment length over Cross-coverage at the read length
- **RIP%** - Percentage of reads wthin peaks
- **RIBL%** - Percentage of reads wthin Blacklist regions

QC Results

Mapping, Filtering and Duplication rate

This section presents the mapping quality, duplication rate and distribution of reads in known genomic features.

Table 2. Number and percantage of mapped,duplicated and MapQ filter passing reads

ID	Tissue	Factor	Condition	Replicate	Unmapped	Mapped	Pass MapQ Filter and Dup	Total Dup%	Pass MapQ Filter%	Pass MapQ Filter and Dup%
22RV1MetR_input	22RV1MetR	H3K27ac	22RV1MetR_input	2	0	4749225	793341	21	86	19
22RV1MetR_IP	22RV1MetR	H3K27ac	22RV1MetR_IP	1	0	4170298	746018	23	78	23
22RV1WT_input	22RV1WT	H3K27ac	22RV1WT_input	2	0	4610655	880691	21	89	21
22RV1WT_IP	22RV1WT	H3K27ac	22RV1WT_IP	1	0	3978495	593468	19	80	19
DU145MetR_input	DU145MetR	H3K27ac	DU145MetR_input	2	0	7519350	1301270	22	77	23
DU145MetR_IP	DU145MetR	H3K27ac	DU145MetR_IP	1	0	9104194	2049872	26	84	27

ID	Tissue	Factor	Condition	Replicate	Unmapped	Mapped	Pass MapQ Filter and Dup	Total Dup%	Pass MapQ Filter%	Pass MapQ Filter and Dup%
DU145WT_input	DU145WT	H3K27ac	DU145WT_input	2	0	7766357	1282783	21	77	22
DU145WT_IP	DU145WT	H3K27ac	DU145WT_IP	1	0	9726192	2104016	25	84	26

Table 2 shows the absolute number of total, mapped, passing MapQ filter and duplicated reads. The percent of mapped reads passing quality filter and marked as duplicates (Non-Redundant Fraction?) are also included.

Description of read filtering and flag metrics:

- **Total Dup%-**Percentage of all **mapped** reads which are marked as **duplicates.**
- **Pass MapQ Filter%-**Percentage of all **mapped** reads which**pass MapQ quality** filter
- **Pass MapQ Filter and Dup%-**Percentage of all reads which pass **MapQ filter** and are marked as**duplicates.**

Duplication rates (Dup %) are dependent on the ChIP library complexity and the number of reads sequenced Higher duplication rates maybe due to low ChIP efficiency when read counts are lower or conversely saturation of ChIP signal when sequencing large number of reads. Since this metric is dependent on both read depth and the properties of the ChIP itself, comparison between biological or technical replicates of similat total read counts can best identify problematic libraries .

Highly mappable (multimappable) positions within the genome can attract large levels of duplication and so assessment of duplication before and after MapQ quality filtering can identify contribution of these positions to the duplication rate.

Figure 1. Heatmap of log2 enrichment of reads in genomic features

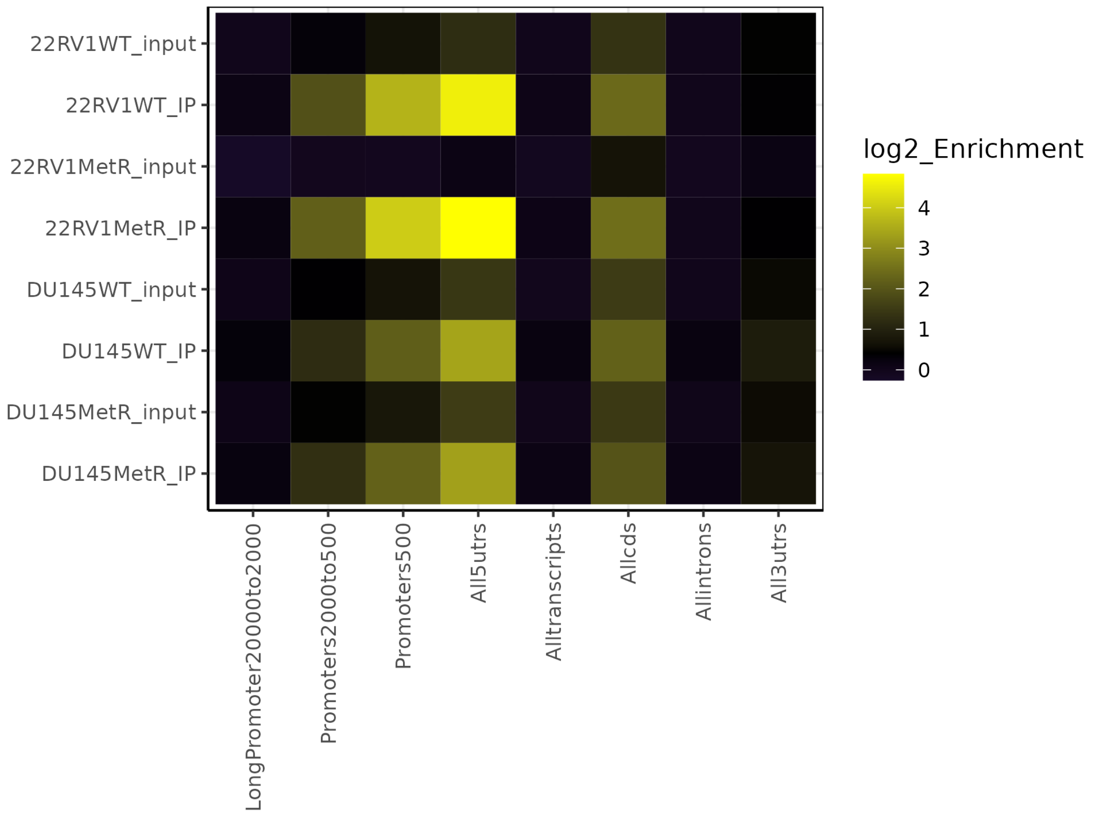

The distribution of reads across known genomic features such as genes and their subcomponents may allow further evaluation of ChIP-seq success and quality. A transcription factor know to preferentially bind at a genomic feature should show relative enrichment against other transcription factors showing no such preference. In addition,a replicate showing a differing enrichment patterns across genomic features compared to those within its sample group would highlight a potential outlier sample worthy of further investigation

Figure 2 shows the log2 enrichment of specified genomic features within samples with regions of greater enrichment showing bright yellow and lower enrichment seen in black

ChIP signal Distribution and Structure

In this section, metrics relating to genome wide depths of coverage and, the relationship between Watson and Crick reads are presented. The metrics are the SSD metric and cross-coverage metrics, Relative_CC and fragmentLength_CC.

Figure 2. Plot of the log2 base pairs of genome at differing read depths

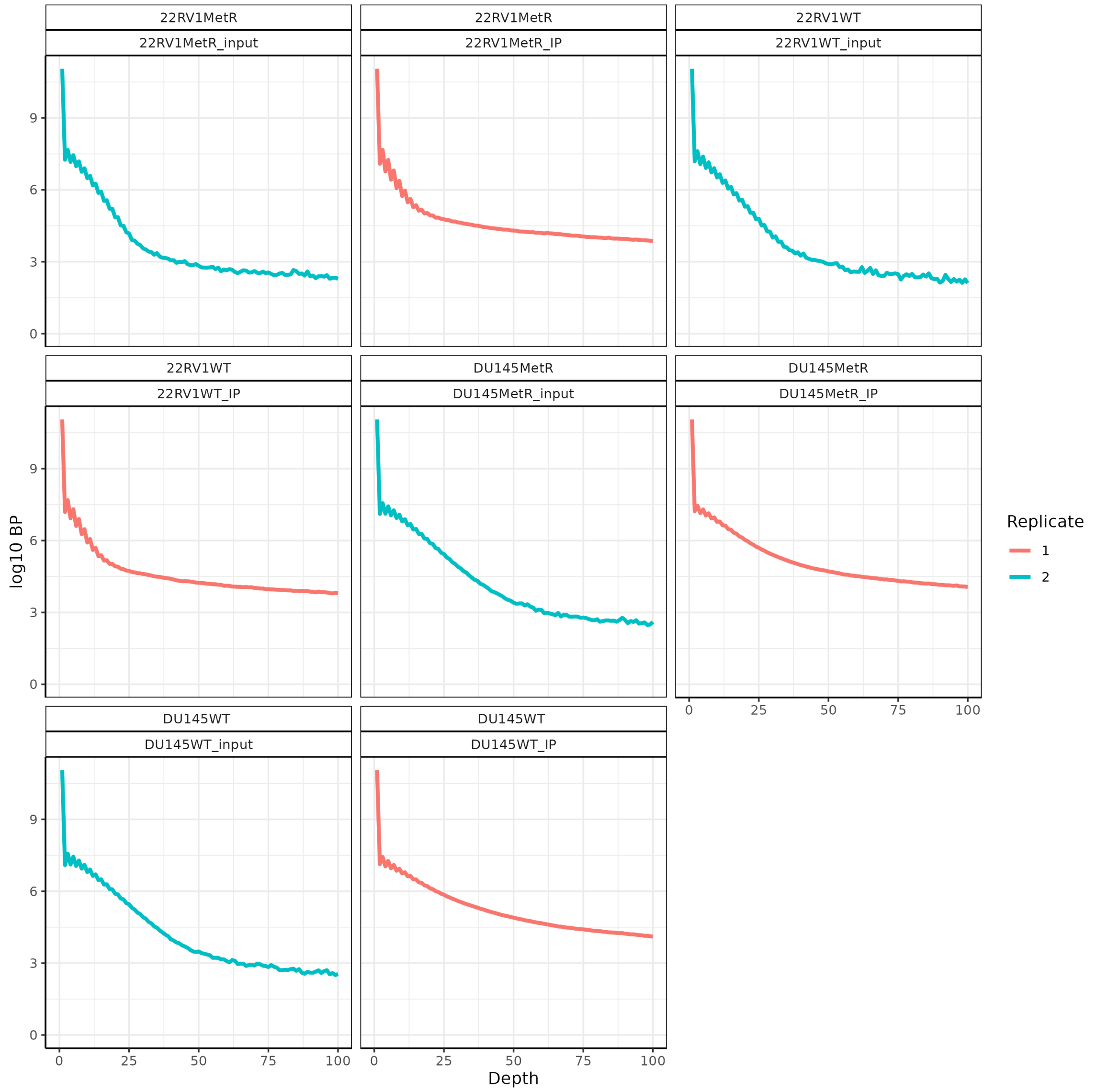

SSD is the standard deviation of coverage normalised to the total number of reads. Evaluation of the number of bases at differing read depths,**(figure 3)**alongside the use of the SSD metric allow for an assessment of the distribution of ChIP-seq or input signal.

Successfull Histone and transcription factor ChIP-seq samples will show a higher proportion of genomic positions at greater depths and equivalence of sample and input SSD scores highlights either an unsuccessful ChIP or high levels of anomalous input signal

Figure 3. Plot of CrossCoverage score after successive strand shifts

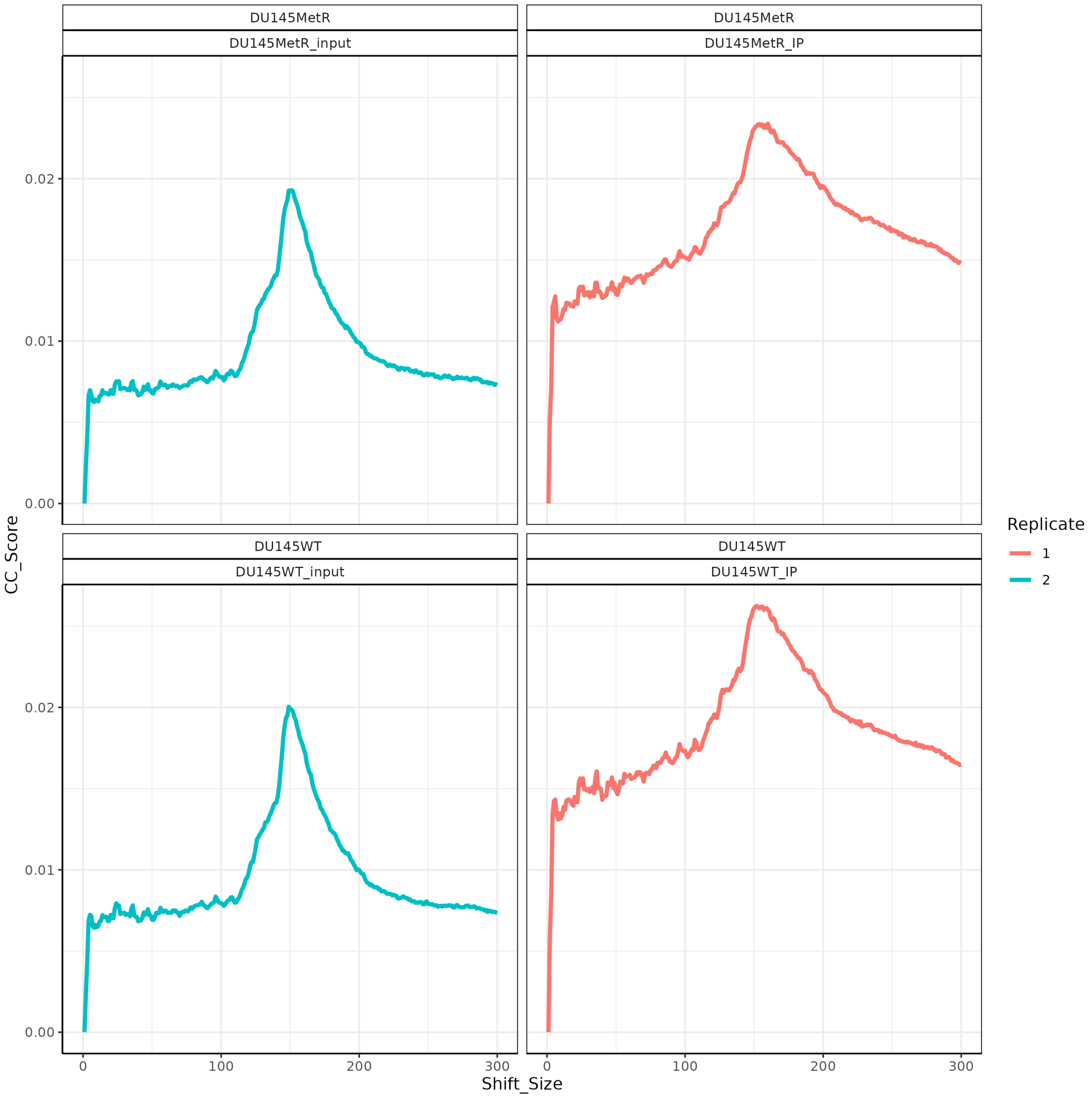

An important measure of ChIP successive is the degree to which Watson and Crick reads cluster around the centres of transcription factor bindind sites or epigentic marks.

Transcription factor binding sites identified by ChIP-seq will show two distinct peaks of Watson and Crick strands separated by the fragment length. Here the method of cross-coverage (ChIPseq package) analysis is used to investigate this spatial clustering of Watson and Crick reads.

To investigate this spatial clustering, reads on the positive strand are shifted in 1bp steps and the total proportion genome now covered by both strands combined is assessed. **Figure 4** shows the CCov_Score (described below) after successive shifts. The points of highest outside of the read-length exclusion region, 2* the read length, (marked in grey) is considered the fragment length

Following the methodology first presented for cross-correlation by Encode to calculate the Relative Strand Cross Correlation (NSC) and Normalised Strand Cross Correlation, the Relative Cross Coverage score and Fragment Length Cross Coverage score are calculated.

The calculation of cross-coverage (CCov),Relative CCov and Fragment Length CCov scores are explained below:

- **CCov_Score-** 1-(Total covered genome size at strand shift)/(covered genome size with no shift)
- **Fragment Length CCov-** (CCov of fragment length strand shift)/(Minimum CCov)
- **Relative CCov-** (CCov of fragment length strand shift)/(CCov of read length strand shift)

Peak Profile and ChIP Enrichment

Following the identification of genome wide enrichment (peak calling), the percentage of ChIP signal within enriched regions, as well the average profile across these regions can be used to further evaluate ChIP quality

Figure 4. Plot of the average signal profile across peaks

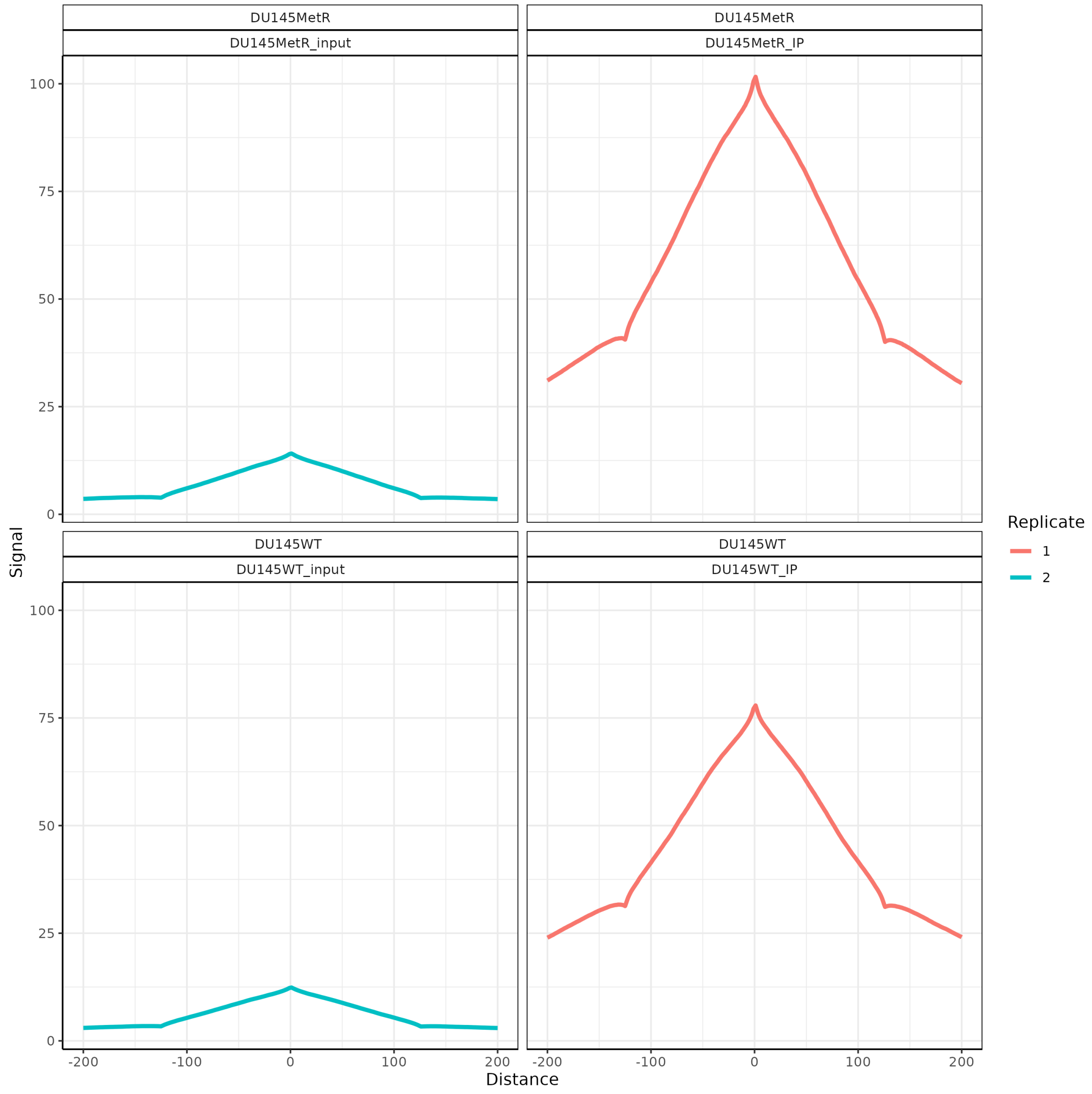

Figure5 represents the mean read depth across and around peaks. By identying the average pattern of enrichment across peaks, differences in both mean peak height and shape may be found. This not only assits in a better characterisation of ChIP enrichment but can aid in the identification of outliers.

Figure 5. Barplot of the percentage number of reads in peaks

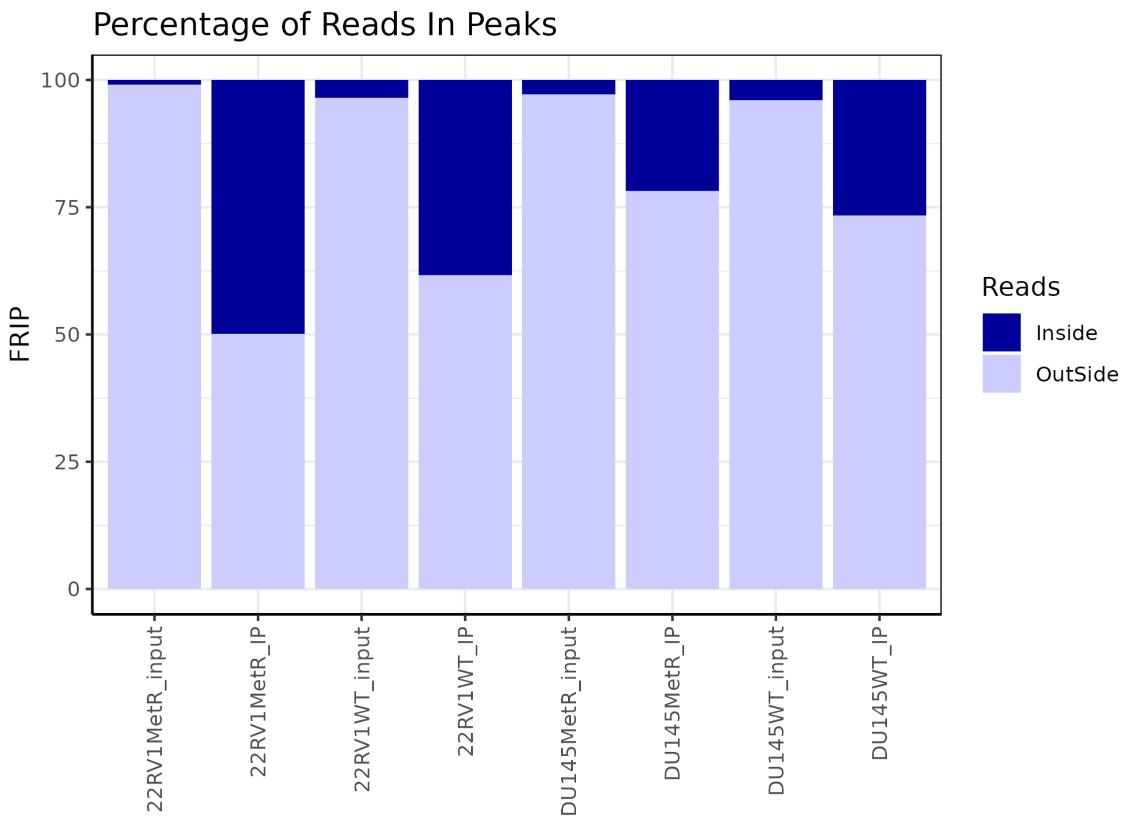

Figure6 shows the total percentage of reads contained within enriched regions or peaks. The higher efficiency ChIP-seq will show a higher percentage of reads in enriched regions/peaks and longer epigenetic marks will often have a higher ranges of efficiencies than punctate marks or transcription factors.

Figure 6. Density plot of the number of reads in peaks

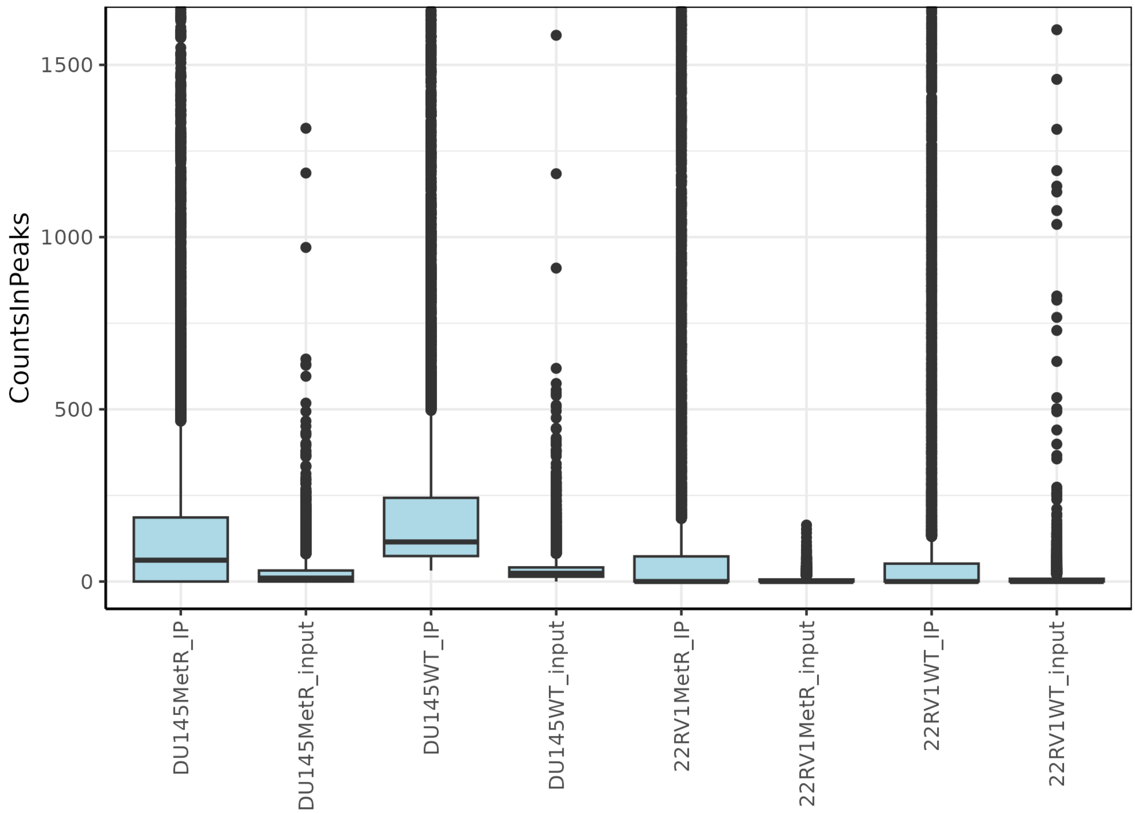

Figure7 shows the distribution of reads in all peaks. Evaluation of the distibution can allow for greater characteriation of the variability and range of signal in peaks within a sample and so better characterise the signal across peaks than the RIP score may allow.

Figure 7. Plot of correlation between peaksets

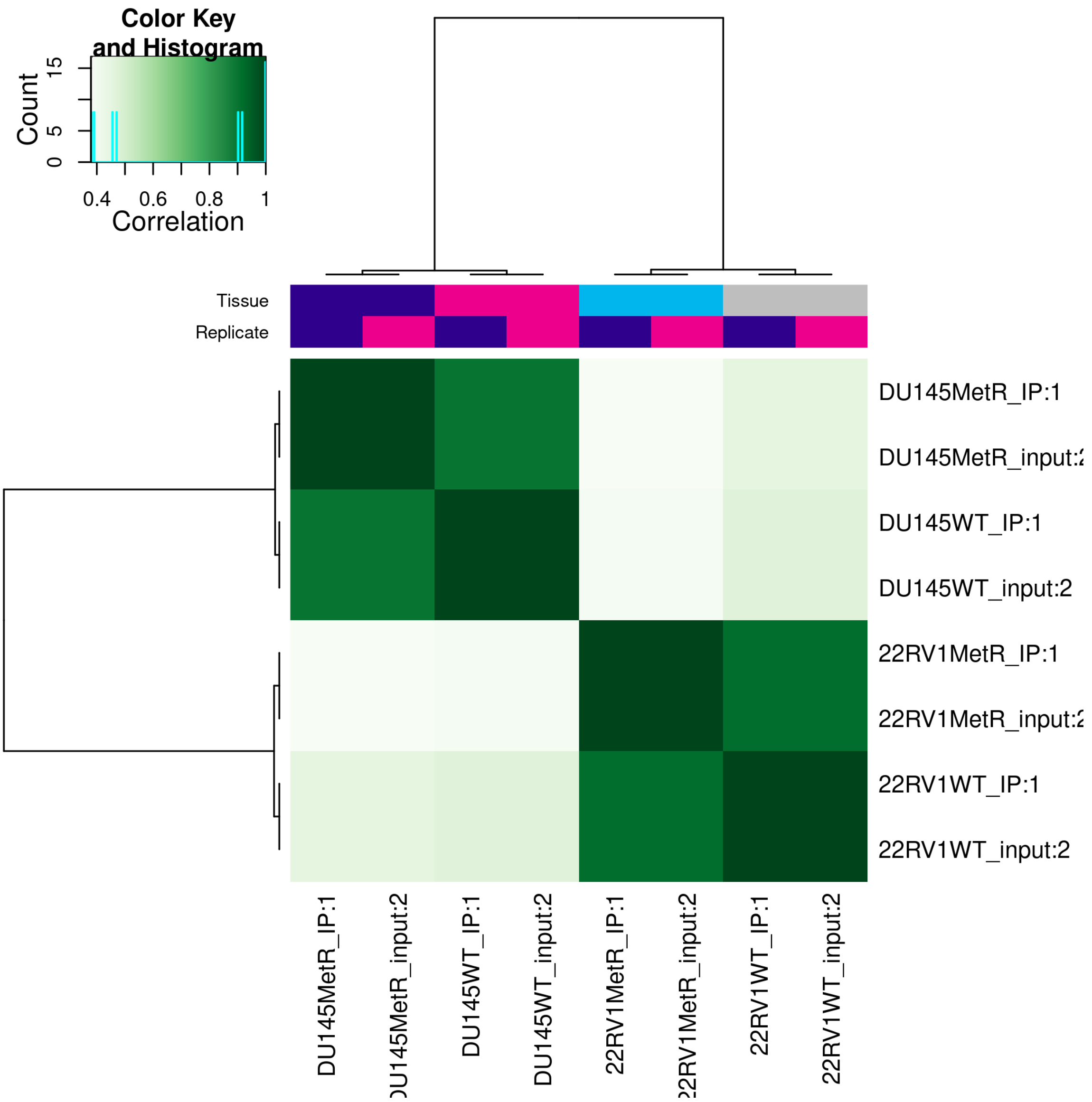

Figure 8. PCA of peaksets

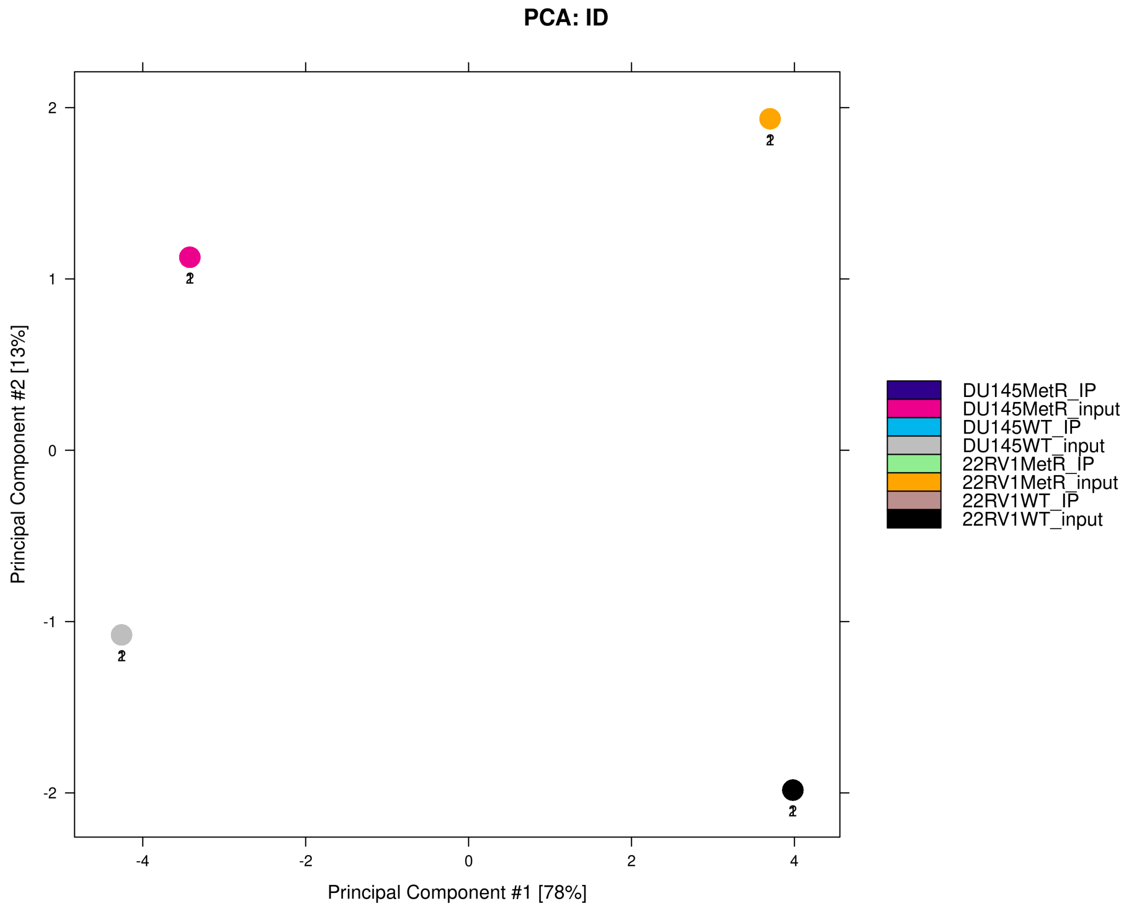

Figure8 and 9 shows the correlation between samples as a heatmap and by principal component analysis. Replicate samples of high quality can be expected to cluster together in the heatmap and be spatially grouped within the PCA plot.

Files and Versions

R Version Information

- Version: 4.2.1
- Version_String :R version 4.2.1 (2022-06-23)

ChIPQC Version Information

- Version: ChIPQC:3.0.0.20230222
- Author: Tom Carroll, Wei Liu, Ines de Santiago, Rory Stark
- Maintainer: Quanhu Sheng , Tom Carroll , Rory Stark

MADE WITH NOZZLE
